# Hybrid Fusion of Time–Intensity Curve, Deep Learning, and Fractional Zernike–Caputo Features for Accurate Liver Lesion Classification in DCE-MRI

**DOI:** 10.3390/jimaging12090415

**Published:** 2026-09-03

**Authors:** Ali M. Hasan, Noor K. N. Al-Waely, Wallaa L. Alfalluji, Rabha W. Ibrahim, Hamid A. Jalab, Farid Meziane

**Affiliations:** 1 College of Medicine, Al-Nahrain University, Baghdad 10001, Iraq; noor83kadhem@nahrainuniv.edu.iq; 2 College of Medicine, Al-Mustaqbal University, Babil 51001, Iraq; wallaa.luay.alfalluji@uomus.edu.iq; 3 Information and Communication Technology Research Group, Scientific Research Center, Al-Ayen University, Nile Street, Thi-Qar 64001, Iraq; rabha@alayen.edu.iq (R.W.I.); hamid.a@alayen.edu.iq (H.A.J.); 4 Data Science Research Centre, School of Computing and Engineering, University of Derby, Derby DE22 3AW, UK; f.meziane@derby.ac.uk

**Keywords:** liver lesion diagnosis, time-intensity curves, deep learning, fractional calculus, fractional Zernike–Caputo, DCE-MRI

## Abstract

Early and accurate detection of liver masses is essential for effective clinical management and improved patient outcomes, as treatment strategies differ significantly between benign and malignant lesions. Dynamic contrast-enhanced magnetic resonance imaging (DCE-MRI) is widely used for liver lesion evaluation due to its ability to capture temporal enhancement behavior. However, reliable interpretation remains challenging because many lesions exhibit similar enhancement patterns, leading to diagnostic uncertainty and potential misclassification. This study proposes a hybrid diagnostic framework that integrates multiple complementary feature representations for automated liver mass classification. Specifically, the model extracts time–intensity curve (TIC) characteristics to capture contrast dynamics, deep learning features to represent complex spatial patterns, and fractional Zernike–Caputo descriptors to encode advanced mathematical shape and texture information. These heterogeneous feature sets are subsequently fused to form a unified and discriminative representation of each lesion. The proposed study aims to enhance the differentiation between benign and malignant liver masses by leveraging the strengths of kinetic, data-driven, and fractional mathematical descriptors. Experimental results demonstrate that the model achieves superior diagnostic performance, reaching an accuracy of 94.05% on a dataset comprising 645 dynamic MRI cases. Overall, the proposed approach provides a robust and efficient tool for liver lesion characterization, with potential to support clinical decision-making and reduce diagnostic ambiguity in medical imaging practice.

## 1. Introduction

Focal liver lesions (FLLs) are common abnormal areas of tissue within the liver, discovered through imaging techniques such as ultrasound (US), computed tomography (CT), or magnetic resonance imaging (MRI). FLLs can be broadly categorized into benign (non-cancerous) and malignant (cancerous) types each with distinct characteristics on medical imaging modalities and requiring precise intervention and an appropriate course of treatment [1]. FLLs may or may not exhibit symptoms and can be brought on by a number of different circumstances. They can be caused by focal nodular hyperplasia, liver cysts, or benign tumors such hepatic adenomas and hemangiomas. Hepatocellular carcinoma and metastatic tumors originating from other organs are examples of malignant tumors. Since the treatment approaches for benign and malignant lesions vary greatly, accurate diagnosis of FLLs is crucial for differentiating between the two. Misdiagnosis can lead to unnecessary interventions for benign lesions or delayed treatment for malignant ones [2,3].

Primary malignant liver tumors commonly develop in patients with underlying liver cirrhosis, with hepatocellular carcinoma (HCC) being the most common primary liver malignancy, while cholangiocarcinoma occurs less frequently. In 2020, according the Global Cancer Observatory, primary liver cancer was ranked as the third most common cause of death among cancer patients [4]. US is widely used as the first-choice imaging modality for patients with suspected liver lesions because of its non-invasive nature, real-time imaging capability, widespread availability, and absence of ionizing radiation. However, US is considered less sensitive and specific than CT and MRI [5]. CT is a useful and efficient medical imaging technique as it provides precise information about the size, shape, and location of liver masses. However, CT scanning may not always detect very small liver lesions due to its spatial resolution that makes it difficult to detect and visualize lesions that are smaller than 1 cm. Furthermore, the use of X-ray exposure in CT scans is undesirable because of the possibility of cell damage and DNA base changes, which raise the incidence of cancer [6]. Furthermore, intravenous iodinated contrast media are often used to further enhance CT scan images. Thus, patients with iodine allergies or with impaired kidney function are put at higher risk of developing contrast-induced adverse effects. Therefore, clinicians may need to use alternative medical imaging methods for liver mass characterization in these patients [7,8].

MRI is considered the preferable tool for assessing liver masses to overcome some of the limitations of US and CT scans in detecting liver lesions, particularly in cases of suspected HCC in patients with cirrhosis or chronic liver disease. Due to its high contrast resolution and more accurate and precise imaging diagnostic, MRI is becoming more popular [9]. It uses a very strong magnetic field and radio frequency electromagnetic waves to create detailed images of the internal organs and tissues of the human body. The combination of radio frequency electromagnetic waves and magnetic field that is generated by gradients results in the production of various MRI pulse sequences including weighted T1, T2, and diffusion, and dynamic contrast enhanced magnetic resonance imaging (DCE-MRI) sequences. Examination usually starts with standard non-enhanced T1- and T2-weighted sequences which provide useful data for the detection and characterization of focal and diffuse liver lesions without contrast enhancement [10]. Furthermore, the use of contrast material in combination with T1-weighted imaging enhances signal intensity, facilitating characterization of focal liver lesions [11]. DCE-MRI is based on implementing acquisition of baseline T1-weighted imaging without gadolinium-based contrast enhancement, followed immediately by a series of T1-weighted images acquired over time during and after gadolinium-based contrast, to distinguish lesions depending on their degree of vascularity and composition.

Time–intensity curves (TICs) are mathematical representations used to characterize lesion enhancement patterns by plotting signal intensity changes over time following contrast agent administration. These curves provide valuable information about the hemodynamic properties within the region of interest (ROI) of a liver lesion. The signal intensity in the liver vessels and certain liver lesions increases significantly, followed by a washout of contrast material due to the diffusion through the interstitial space, which occurs at a rate in the order of 50 to 100 μm/second [12]. Since there are no set rules for ROI placement during image processing, an expert radiologist should carefully position the ROI over the area showing the earliest and largest contrast in any dynamic series [6,13].

Thus, TIC curves include two phases. The initial upslope includes the first 2 min of DCE-MRI until the first change in the curve. Then, the delayed phase starts, which can be Type 1 (progressive enhancement), Type 2 (plateau), or Type 3 (washout) according to suspicious lesions. Type 1 demonstrates a continuous increase in the signal intensity after contrast injection in the delayed phase of DCE-MRI. This pattern is known by homogeneous enhancement and is commonly seen in 83% of benign lesions and 9% of malignant lesions [14]. When the signal intensity remains relatively stable during the delayed phase after an initial sharp rise, this enhancement pattern is Type 2, which is often noted to be common in several benign liver lesions and some malignant liver lesions [15]. Type 3 exhibits a rapid decrease in signal intensity during the delayed phase after a quick rise in the initial phase. This pattern may be seen in malignant liver lesions which show heterogeneous enhancement [6,16].

Previous studies have shown that liver lesion kinetic curves provide high sensitivity but limited specificity in distinguishing benign from malignant lesions. In general, malignant lesions are characterized by rapid enhancement followed by washout, while benign lesions exhibit diverse enhancement profiles, including persistent enhancement, plateau patterns, and occasional washout. This results in a significant overlap with malignant lesions. Furthermore, in clinical practice, the interpretation of the DCE-MR images is subjective and depends on the recognition of signature enhancement patterns that have been described for various focal liver lesions. The interpretation is subjective and prone to interobserver errors [17].

This study aims to enhance the diagnosis of benign and malignant liver lesions by combining the time–intensity patterns of contrast agent uptake in liver tissue with radiomic features, thereby reducing reliance on invasive biopsy procedures. Liver biopsy remains a standard diagnostic approach for confirming malignancy. However, it carries potential risks such as pain at the biopsy site and post-procedural bleeding and in some extreme cases, bacterial infection may occur [18,19].

Recently, many studies have investigated the vital role of kinetic models in improving the diagnosis of liver lesions by analyzing changes over time and helping to differentiate benign and malignant lesions precisely. Using long-range contrast-enhanced ultrasound (CEUS) images and clinical variables, Liu, Tang [20] created a deep learning (DL) radiomics model and examined its efficacy in diagnosing FLLs. The highest achieved area under the curve (AUC) was 96.6% in diagnosing a dataset of 303 patients. Nayantara, Kamath [21] proposed an automated system using a DL model based on SegNet and an atrous spatial pyramid pooling module to segment liver cancer. This was followed by feature extraction using histograms, wavelets, and DL to classify liver cancers into benign, malignant and metastatic tumors. The averaged achieved accuracy was 81.9% for the utilized public dataset of 601 retrospective cases, using the support vector machine (SVM) classifier. Zossou, Gnangnon [22] used radiomics features to differentiate abnormal tissue from healthy liver tissue. The first-order, second-order, higher-order, and shape statistics were used to extract 1686 features from the regions of interest of each DCE-MRI slice. The extracted features were refined statistically via the Student’s *t*-test and lasso regression. The best score was achieved by naïve Bayes with an area under the curve (ROC) varying from 59.3% to 92.6% when classifying a dataset that included 94 DCE-MRI liver scans. Zhou, Wang [23] proposed an automated framework based on hierarchical convolutional neural networks (CNNs) for detecting and classifying FLLs in CT scans into three types of malignant and benign lesions. The averaged achieved accuracy was 82.5% on a dataset that included 616 liver CT scans. Du, Yuan [24] compared the performance of a DL model with a radiomics model for differentiating liver tumor grades based on DCE-MRI scans. The deep features were extracted by using a DenseNet model, while the radiomics model was constructed by using pre-defined features and SVM. The achieved AUC was 85.7% and 83.8% via the radiomics model and deep learning model, respectively. In order to distinguish dual-phenotype hepatocellular carcinoma (DPHCC) from HCC and intrahepatic cholangiocarcinoma (ICC), Wu, Zhang [25] created an automated approach to verify radiomics and deep learning models based on DCE-MRI liver scans. In that study, the combination of radiomics and deep features showed competent diagnostic performance with an AUC of 99% to classify a dataset consisting of 381 liver DCE-MRI scans. Based on previous studies, developing and validating an automated system for differentiating malignant liver lesions from DCE-MRI sequences could assist clinicians in reducing the need for invasive biopsy procedures prior to surgery. The relatively large cohort of 645 cases provides a substantial dataset for model development and evaluation and represents an important strength of the present study. However, the primary methodological contribution of the proposed framework lies in the integration of complementary kinetic, fractional Zernike–Caputo (FZC), and deep learning features to improve the characterization and classification of focal liver lesions. By combining time–intensity curve (TIC) analysis, deep learning features, and fractional Zernike–Caputo descriptors, the proposed framework aims to provide more comprehensive and discriminative feature representations for differentiating benign and malignant liver lesions. The following is a summary of this study’s contributions:

1. Novel hybrid diagnostic framework:

This study proposes an integrated model that combines time–intensity curve (TIC) analysis, deep learning features, and fractional Zernike–Caputo descriptors for comprehensive liver lesion characterization from DCE-MRI.

2. Multi-source feature fusion strategy:

A unified representation is developed by fusing kinetic (TIC), data-driven (deep learning), and fractional mathematical features, which is intended to provide a more discriminative representation for classification between benign and malignant lesions.

3. Incorporation of fractional descriptors:

The use of fractional Zernike–Caputo features introduce advanced shape and texture characterization, capturing subtle patterns that are often missed by conventional methods.

4. Reduction of invasive procedures:

Improving non-invasive diagnostic accuracy may enhance the characterization of focal liver lesions and potentially reduce the need for invasive diagnostic procedures.

## 2. Materials and Methods

**Figure 1:**
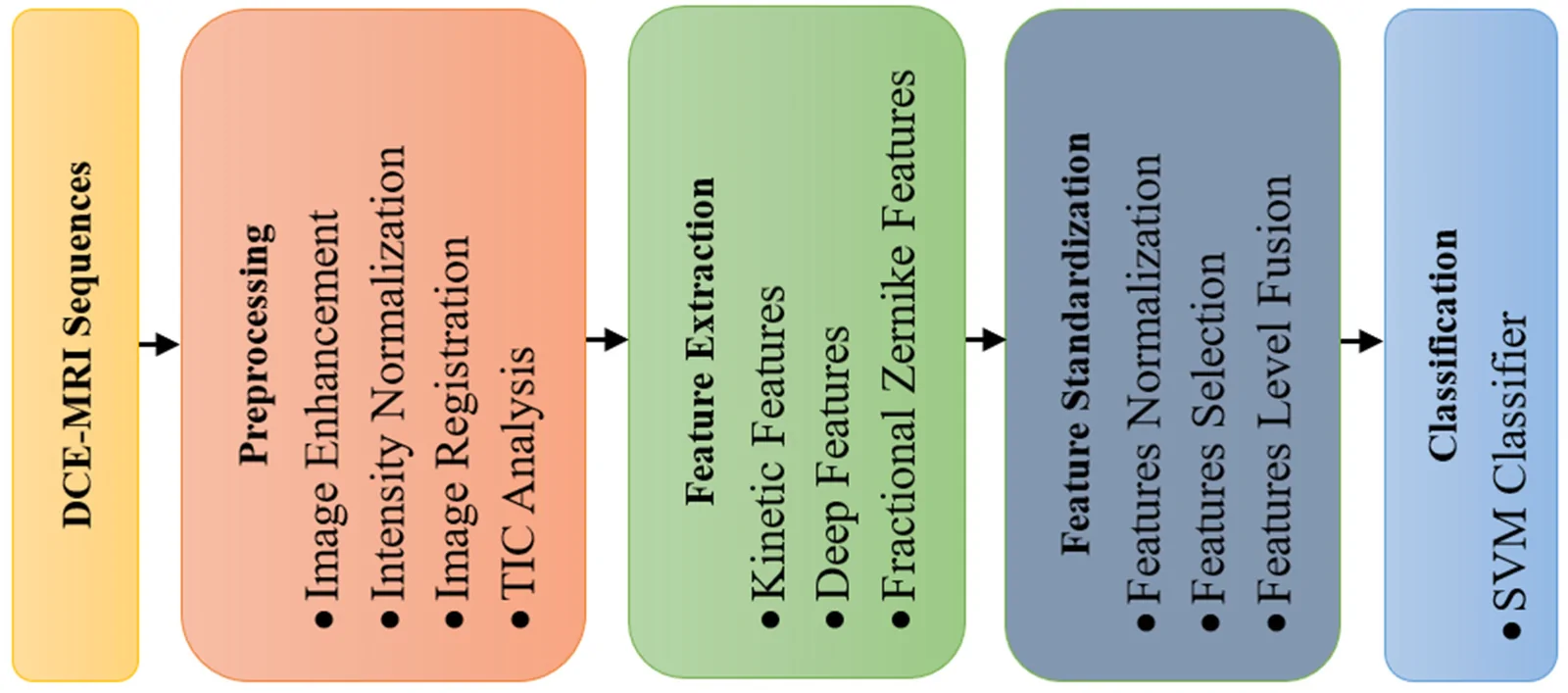
Proposed Model.

This study integrates diverse features extracted from dynamic contrast-enhanced MRI of the liver to develop a more accurate diagnostic tool for differentiating benign from malignant liver lesions, potentially reducing the need for invasive follow-up procedures such as biopsies. The proposed study comprises four stages, starting from the data collection and clinical diagnosis, preprocessing, feature extraction, and classification. The flow process is illustrated in Figure 1.

### 2.1. Dataset

The utilized DCE-MRI liver scans used in this study were collected from January 2023 to September 2025 in Buratha Diagnostic Imaging Center, Iraq. The data was anonymized and patients where the clinical diagnosis did not correspond with the pathological re-sults were excluded from the study. Additionally, individuals receiving any disease-related treatment before taking the test were excluded; this included systemic chemotherapy, transarterial chemoembolization therapy, radiofrequency ablation, and surgery. Furthermore, patients with a history of liver resection or liver transplantation were also excluded, and poor-quality images were excluded.

According to the established selection criteria, a dataset comprising images from 645 patients was assembled. Among these, 350 (54.26%) cases were classified as malignant, based on histopathological examination, or, when pathological confirmation was unavailable, on the integration of characteristic radiological findings with relevant clinical information. The remaining 295 cases (45.73%) were diagnosed as benign. The distribution of lesions according to the number of lesions per patient, anatomical location, and lesion size was also evaluated. The numbers of patients with one lesion and multiple lesions were 553 and 92, respectively. Among the patients with multiple lesions, 74 patients had two lesions and 18 patients had three lesions.

Additionally, patients were categorized by lesion location, with right-lobe, left-lobe, and bilobar lesions accounting for 626 (82.9%), 111 (14.7%), and 18 (2.4%) cases, respectively. The overall lesion size ranged from 10 to 177 mm, with a mean value of 61.80 ± 40.88 mm and a median value of 54 mm. Benign lesions had a mean size of 48.88 ± 32.82 mm (range, 10–107 mm), whereas malignant lesions had a mean size of 70.96 ± 44.12 mm (range, 14–177 mm).

**Figure 2:**
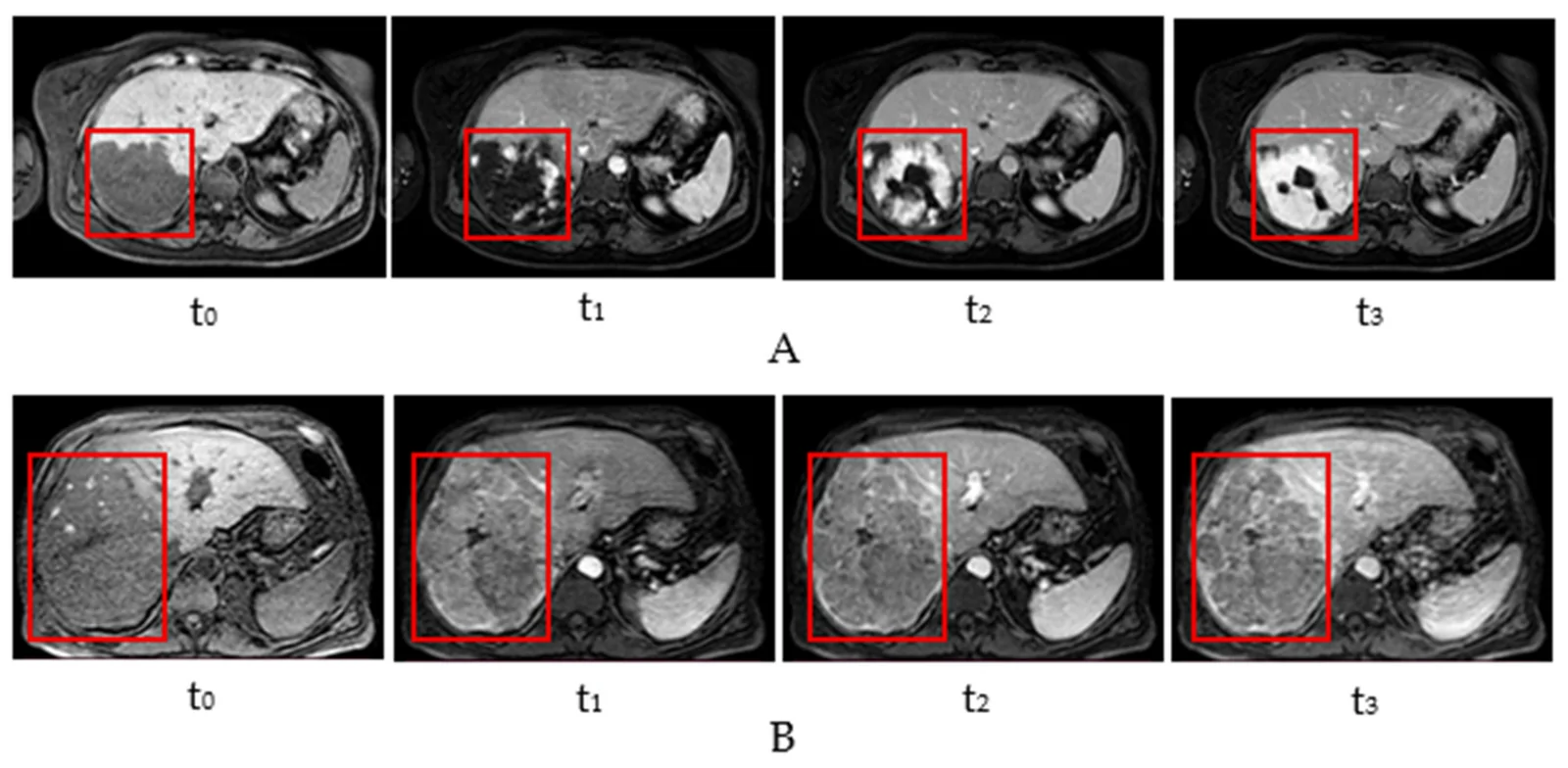
DCE-MRI scans of two patients with FLL inside the red rectangles. (**A**) Patient with benign FLL lesion, and (**B**) Patients with malignant FLL lesions.

MRI examinations were performed using a 1.5-T Philips Achieva MRI scanner (Philips Healthcare, Best, The Netherlands) with a 16-channel Torso XL radiofrequency coil. For DCE-MRI, Gadovist (gadolinium-based contrast agent) was administered at a dose of 2 mL/kg. Following intravenous contrast administration, four consecutive dynamic acquisitions were performed. Each dynamic acquisition required approximately 17 s, resulting in a total dynamic acquisition time of approximately 68 s (1 min 8 s). The four dynamic acquisitions were subsequently used for the kinetic analysis. Figure 2 shows two DCE-MRI scans from the collected dataset.

### 2.2. DCE-MRI Liver Preprocessing

Numerous artefact types occur during MRI scans, leading to degraded image quality and potentially affecting the diagnostic accuracy by being confused with a pathology. These artifacts can be caused by various factors such as patient motion, implant metal, or machine error. Furthermore, significant intensity variations in MRI scans may occur due to inter-scan and intra-scan factors, which makes direct image comparison difficult across different imaging scanners and even within the same subject over time [26]. To reduce imaging variability and improve data quality for subsequent analysis, liver DCE-MRI images were preprocessed using Gaussian filtering followed by histogram normalization.

The Gaussian filter was employed to smooth the images by reducing noise and eliminating undesired high-frequency components while preserving the overall anatomical structures. Following noise suppression, histogram normalization was applied to standardize intensity distributions and enhance image contrast. The Gaussian filter operated as a spatial low-pass filter in which neighboring pixels were weighted according to a Gaussian distribution characterized by the variance parameter σ. The corresponding Gaussian function $g\left(x,y\right)$ is expressed as:

$$g\left(x,y\right)=e^{\left(\frac{-(x^{2}+y^{2})}{2\sigma^{2}}\right)}$$

where x and y are the pixel’s coordinates in the DCE-MRI image, and σ is the variance that controls the degree of smoothing.

Furthermore, collecting a dataset from different MRI scanners with different pixel spacing and spatial resolutions contributes to significant statistical changes in the MR images. Therefore, all liver DCE-MRI scans were adjusted by histogram normalization to stretch the grey-level range from 0 to 255 using the following linear transformation algorithm in order to reduce variations resulting from different image acquisition protocols in MRI scanners [27]:

$$im^{’}\left(x,y\right)=\frac{\left(im\left(x,y\right)-\min\left(im\right)\right)}{\left(\max\left(im\right)-min(im)\right)}\times 255$$

where im and $im^{’}$ indicate the original and normalized DCE-MRI scan, respectively, and min and max represent the minimum and maximum signal intensity values of the pixels within that DCE-MRI scan. Furthermore, the liver lesions and TIC, respectively, were manually extracted and analyzed qualitatively with Radiant DICOM viewer 2025.2 software. The ROI within the liver lesion that showed the most enhancement across all imaging sequences was defined by an expert radiologist in a clinical setting [6,28]. Diffuse liver lesions were excluded from this study because their characteristics are not specific enough for precise diagnosis by drawing the TIC. Additionally, misregistration artifacts caused by respiratory motion were also excluded because these artifacts significantly impact on reliability of data used to draw the TIC by blurring images and altering apparent contrast dynamics [17]. The gathered liver DCE-MRI scans were then divided into three kinetic pattern curves based on the generated TIC curves. The histological confirmation for distinguishing benign from malignant liver lesions was connected with these kinetic patterns.

### 2.3. Feature Extraction

Feature extraction included the process of transforming the raw data of the liver DCE-MRI into more informative set of attributes to enhance diagnostic efficiency for liver diseases.

- Time–Intensity Features

Time–intensity features describe the temporal behavior of tissue enhancement on liver DCE-MRI imaging, providing valuable insight into underlying vascularity and perfusion of liver cancer. Malignant liver tissues typically exhibit rapid enhancement followed immediately by early washout due to abnormal angiogenesis and increased vascular permeability, unlike benign liver tissues, which usually exhibit slow, progressive, and sustained enhancement rather than rapid washout. Contrasting, these dynamic characteristics of malignant and benign liver cancer, several quantitative measures can be derived from the TIC, such as signal intensity slopes, maximum signal intensity, initial enhancement, peak enhancement, early signal enhancement ratio, second enhancement, and the gradient [6,12,29]. These kinetic SI measures can contribute to improving diagnostic confidence with regard to liver cancer and provide better treatment planning in clinical and research settings [6,12,29,30,31].

B. Deep Learning Feature Extraction

Although kinetic curves from DCE-MRI sequences provide valuable information about liver cancer vascularity, they are not entirely specific and overlap can occur between benign and malignant liver cancers. This represents the main limitation of utilizing the kinetic curve in the diagnosis of liver cancer [32]. Therefore, a learning-based method is required to extract high-level spatiotemporal characteristics from liver DCE-MRI scans to overcome the limitations of hand-crafted TIC features. This approach has been successfully applied in various fields of science and technology [12,33]. In this study, the liver DCE-MRI scans were treated as a sequential input modeled through two-dimensional-CNN, where the convolutional layers captured hierarchical features from the intensity and enhancement patterns, and discriminative characteristics such as intra-lesional texture, lesion margin, and heterogeneity texture features.

To ensure accurate deep-feature extraction from liver DCE-MRI sequences (t_0_, t_1_, t_2_, and t_3_ respectively), each liver lesion was cropped manually by an experienced radiologist based on the maximum dimension of each lesion prior to the implementation of the CNN. All cropped images were standardized to 113 × 141 pixels. The proposed CNN contains a set of components including convolutional layers, pooling layers, activation functions, and fully connected layers, each with a distinct role in transforming the DCE-MRI slices into the desired output after training. Each convolutional layer includes a set of convolution kernels known as kernel filters. These kernels are small matrices that slide across the DCE-MRI slice, computing the dot product at each position to produce a feature map that highlights specific patterns or features in the given DCE-MRI slice. These kernels move by a number of rows/columns at a time in the convolutional layer, in a process called the stride. Thus, the spatial resolution progressively decreases after every layer. Then, a feature map is produced by applying a convolutional kernel filter, which includes negative and positive numbers, representing the features detected by the filter. Thereafter, this feature map is rectified by implementing ReLU activation function to preserve positive values and set all the negative values to zero. Then, a pooling layer is used to execute the down-sampling on the rectified feature map. The max-pooling function is used to determine the maximum number in every sub-region of the rectified feature maps [12]. The CNN training process is then regularized by normalizing the corrected feature map using a batch normalization layer. Finally, the weights of CNN are updated and improved iteratively through the minimization of a gradient-based optimization algorithm within the training process [33,34].

**Figure 3:**
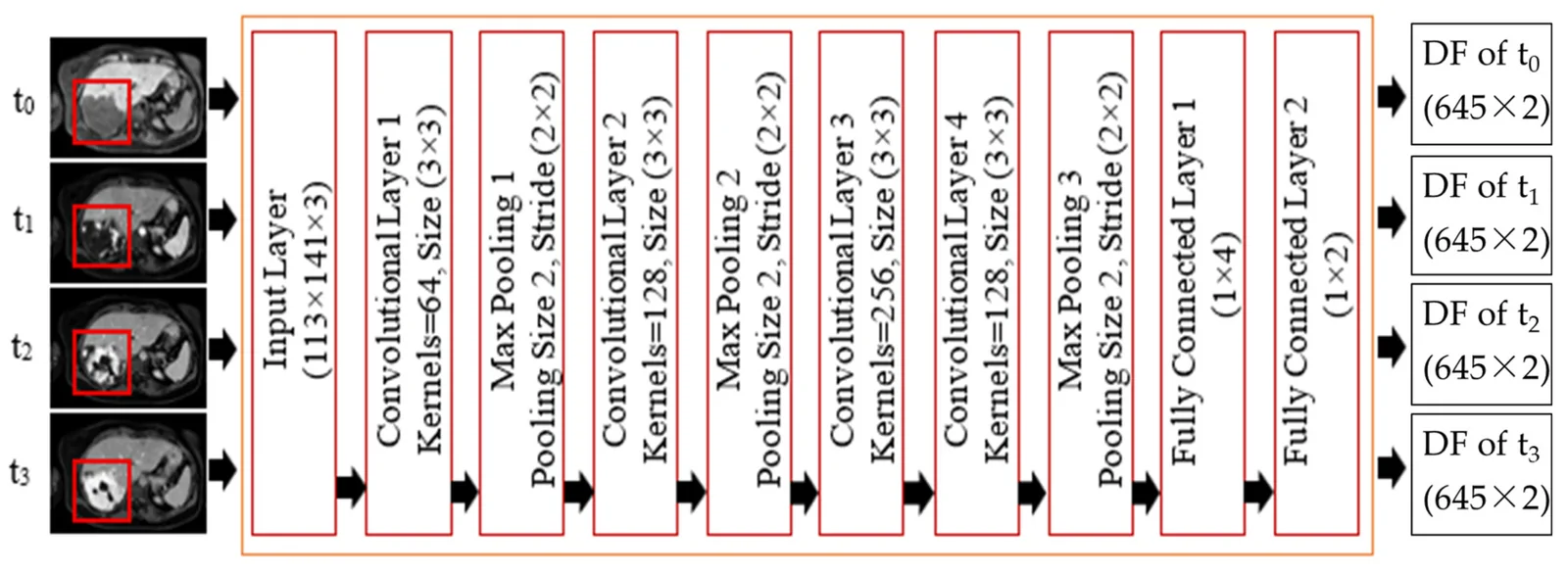
Proposed structure for deep-feature extraction from liver DCE-MRI sequences. The red squares indicate the regions of interest (ROIs) selected from the DCE-MRI images and used as input to the deep-feature extraction network.

The efficiency of a CNN architecture depends significantly on how the convolutional layers are interconnected and how the appropriate weights are set. The proposed CNN architecture includes four convolutional layers, three pooling layers, and two fully connected layers, as demonstrated in Figure 3. The recognized ROI images from dynamic contrast MRI of the liver (t_0_, t_1_, t_2_, and t_3_) are fed independently to the proposed CNN to extract four deep-feature subsets of size (645 × 2).

C. Origin-Regular Generalized Fractional-Degree Zernike–Caputo Radial Features

Zernike polynomials form a classical system for representing functions defined on the unit disk $D=\left\{\left(\rho ,\theta \right):0\leq \rho \leq 1,\;0\leq \theta <2\pi \right\}.$ Their radial structure makes them useful for describing the internal intensity, shape, and texture variations of a normalized image region. For whole-region image-feature extraction, the radial kernel should remain bounded at the center of the disk. We therefore adopt an origin-regular generalization and apply the standard left-sided Caputo derivative with lower terminal zero. For whole-region image-feature extraction, the origin-regular generalized fractional-degree Zernike radial function is adopted. This choice guarantees bounded behavior at the center of the normalized image disk and permits the use of the standard left-sided Caputo derivative with lower terminal zero. The boundary-normalized formulation is more appropriate for contour-specific or annular analysis, since a genuinely non-integer degree may produce singular behavior at the origin. Accordingly, the origin-regular construction is used for the primary internal-texture features, while the boundary-normalized construction may be employed separately as a supplementary descriptor of edge and contour characteristics.

- Classical Zernike radial polynomials

Let $n,m\in N_{0}$ satisfy $0\leq m\leq n,\;n-m\in 2N_{0}.$ The classical Zernike radial polynomial is defined as follows:

(1) $$R_{n}^{m}\left(\rho \right)=\sum_{s=0}^{\left(n-m\right)/2}{\left(-1\right)}^{s}\frac{\left(n-s\right)!}{s!\left(\frac{n+m}{2}-s\right)!\left(\frac{n-m}{2}-s\right)!}\rho^{\;n-2s},\;\;0\leq \rho \leq 1.$$

It satisfies $R_{n}^{m}\left(1\right)=1$ and has the origin behavior $R_{n}^{m}\left(\rho \right)=O\left(\rho^{m}\right),\;\rho \to 0^{+}.$ Writing $N=\frac{n-m}{2},$ the radial polynomial can equivalently be represented as follows:

(2) $$R_{m+2N}^{m}\left(\rho \right)={\rho^{m}}_{2}F_{1}\left(-N,N+m+1;1;1-\rho^{2}\right).$$

It also admits the origin-centered representation:

(3) $$R_{m+2N}^{m}\left(\rho \right)={\left(-1\right)}^{N}\frac{{\left(m+1\right)}_{N}}{N!}{\rho^{m}}_{2}F_{1}\left(-N,N+m+1;m+1;\rho^{2}\right),$$

where ${\left(q\right)}_{k}=\frac{\Gamma \left(q+k\right)}{\Gamma \left(q\right)}$ denotes the Pochhammer symbol. The corresponding complex Zernike mode is:

(4) $$V_{n}^{m}\left(\rho ,\theta \right)=R_{n}^{\left|m\right|}\left(\rho \right)e^{im\theta },\;\;-n\leq m\leq n.$$

In real form, one may use:

(5) $$V_{n,m}^{c}\left(\rho ,\theta \right)=R_{n}^{m}\left(\rho \right)cos\left(m\theta \right),\;\;V_{n,m}^{s}\left(\rho ,\theta \right)=R_{n}^{m}\left(\rho \right)sin\left(m\theta \right).$$

For a fixed azimuthal order m, the radial polynomials satisfy the orthogonality relation:

(6) $$\int_{0}^{1}R_{n}^{m}\left(\rho \right)R_{n’}^{m}\left(\rho \right)\;\rho \;d\rho =\frac{\delta_{nn’}}{2\left(n+1\right)}.$$

- Standard Caputo fractional derivative

Let 0<α<1 and let f be absolutely continuous on $\left[0,1\right]$. The standard left-sided Caputo fractional derivative with lower terminal zero is defined as follows:

(7) $$D_{0+}^{\alpha ,C}f\left(\rho \right)=\frac{1}{\Gamma \left(1-\alpha \right)}\int_{0}^{\rho }{\left(\rho -s\right)}^{-\alpha }f’\left(s\right)\;ds.$$

For a positive power β>0,

(8) $$D_{0+}^{\alpha ,C}\rho^{\beta }=\frac{\Gamma \left(\beta +1\right)}{\Gamma \left(\beta +1-\alpha \right)}\rho^{\beta -\alpha }.$$

The constant case must be treated separately:

(9) $$D_{0+}^{\alpha ,C}1=0.$$

Thus, Equation (8) must not be applied to β=0 when the Caputo derivative is used.

- Origin-regular generalized fractional-degree radial function

Let $m\in N_{0},\;\nu \in R,\;\nu \geq m,$ and define $a_{\nu ,m}=\frac{m-\nu }{2},\;b_{\nu ,m}=\frac{m+\nu }{2}+1$.

**Definition** **1.** *The origin-regular generalized fractional-degree Zernike radial function is defined as follows:*

(10) $${\tilde{R}}_{\nu }^{m}\left(\rho \right)={\rho^{m}}_{2}F_{1}\left(\frac{m-\nu }{2},\frac{m+\nu }{2}+1;m+1;\rho^{2}\right),\;\;0\leq \rho <1.$$

The hypergeometric expansion of (10) is:

(11) $${\tilde{R}}_{\nu }^{m}\left(\rho \right)=\sum_{k=0}^{\infty }A_{k}\left(\nu ,m\right)\rho^{m+2k},\;\;0\leq \rho <1,\;A_{k}\left(\nu ,m\right)=\frac{{\left(\frac{m-\nu }{2}\right)}_{k}{\left(\frac{m+\nu }{2}+1\right)}_{k}}{{\left(m+1\right)}_{k}\;k!}.$$

Since $A_{0}\left(\nu ,m\right)=1$, it follows that

(12) $${\tilde{R}}_{\nu }^{m}\left(\rho \right)=\rho^{m}\left[1+O\left(\rho^{2}\right)\right],\;\;\rho \to 0^{+}\;{\tilde{R}}_{\nu }^{m}\left(0\right)=\left\{\begin{array}{ll} 1, & m=0,\\ 0, & m\geq 1. \end{array}\right.$$

This origin regularity is essential for whole-region image analysis because the image center corresponds to ρ=0.

- Reduction to the classical radial polynomial

Suppose that $\nu =m+2N,\;N\in N_{0}.$ Then $\frac{m-\nu }{2}=-N,$ and the hypergeometric series terminates. Therefore,

(13) $${\tilde{R}}_{m+2N}^{m}\left(\rho \right)=\rho^{m}\sum_{k=0}^{N}\frac{{\left(-N\right)}_{k}{\left(N+m+1\right)}_{k}}{{\left(m+1\right)}_{k}\;k!}\rho^{2k}.$$

Comparison with (3) gives the following:

(14) $${\tilde{R}}_{m+2N}^{m}\left(\rho \right)={\left(-1\right)}^{N}\frac{N!}{{\left(m+1\right)}_{N}}R_{m+2N}^{m}\left(\rho \right).$$

Equivalently,

(15) $$R_{m+2N}^{m}\left(\rho \right)={\left(-1\right)}^{N}\frac{{\left(m+1\right)}_{N}}{N!}{\tilde{R}}_{m+2N}^{m}\left(\rho \right).$$

Thus, the origin-regular generalized function recovers the classical Zernike radial polynomial up to a nonzero normalization constant.

- Fractional Zernike–Caputo radial function

The Caputo derivative can now be applied to the origin-regular series because every term is regular at the lower terminal.

**Proposition** **1.** *Let* $m\in N_{0},\;\nu \in R,\;0<\alpha <1$*. For* 0<ρ<1*, the Caputo derivative of the origin-regular generalized fractional-degree Zernike radial function is as follows:*

(16) $$\begin{array}{rr} D_{0+}^{\alpha ,C}{\tilde{R}}_{\nu }^{m}\left(\rho \right)= & \sum_{\begin{array}{c} k\geq 0\\ m+2k>0 \end{array}}A_{k}\left(\nu ,m\right)\frac{\Gamma \left(m+2k+1\right)}{\Gamma \left(m+2k+1-\alpha \right)}\rho^{m+2k-\alpha }. \end{array}$$

*Equivalently,*

(17) $$\begin{array}{c} D_{0+}^{\alpha ,C}{\tilde{R}}_{\nu }^{m}\left(\rho \right)=\sum_{\begin{array}{c} k\geq 0\\ m+2k>0 \end{array}}\frac{{\left(\frac{m-\nu }{2}\right)}_{k}{\left(\frac{m+\nu }{2}+1\right)}_{k}}{{\left(m+1\right)}_{k}\;k!}\\ \times \frac{\Gamma \left(m+2k+1\right)}{\Gamma \left(m+2k+1-\alpha \right)}\rho^{m+2k-\alpha }. \end{array}$$

*The condition* m+2k>0 *removes only the constant term corresponding to* m=0 *and* k=0*.*

**Proof.**  Fix $r\in \left(0,1\right)$. The hypergeometric series in (11) and its ordinary derivative converge uniformly on the compact interval $\left[0,r\right]$. Hence, the series may be differentiated and substituted term by term into the Caputo integral. For every term satisfying m+2k>0, Equation (8) gives the following:

(18) $$D_{0+}^{\alpha ,C}\rho^{m+2k}=\frac{\Gamma \left(m+2k+1\right)}{\Gamma \left(m+2k+1-\alpha \right)}\rho^{m+2k-\alpha }.$$

If m=0 and k=0, the corresponding term is $A_{0}\left(\nu ,0\right)\rho^{0}=1,$ whose Caputo derivative is zero by (9). Therefore,

$$D_{0+}^{\alpha ,C}{\tilde{R}}_{\nu }^{m}\left(\rho \right)=\sum_{\begin{array}{c} k\geq 0\\ m+2k>0 \end{array}}A_{k}\left(\nu ,m\right)\frac{\Gamma \left(m+2k+1\right)}{\Gamma \left(m+2k+1-\alpha \right)}\rho^{m+2k-\alpha }.$$

Substitution of the coefficient formula proves Equation (17).

□

**Definition** **2.** *The generalized fractional-degree Zernike–Caputo radial feature is defined as follows:*

(19) $$Z_{\nu }^{m,\alpha }\left(\rho \right):=D_{0+}^{\alpha ,C}{\tilde{R}}_{\nu }^{m}\left(\rho \right),\;\;0<\rho <1.$$

The corresponding complex angular mode is:

(20) $$V_{\nu }^{m,\alpha }\left(\rho ,\theta \right)=Z_{\nu }^{\left|m\right|,\alpha }\left(\rho \right)e^{im\theta }.$$

Its real components can be written as follows:

(21) $$\begin{array}{rr} V_{\nu ,m}^{c,\alpha }\left(\rho ,\theta \right) & =Z_{\nu }^{m,\alpha }\left(\rho \right)cos\left(m\theta \right),\\ V_{\nu ,m}^{s,\alpha }\left(\rho ,\theta \right) & =Z_{\nu }^{m,\alpha }\left(\rho \right)sin\left(m\theta \right). \end{array}$$

- Behavior of the Caputo-transformed radial feature

For m≥1, the leading term of Equation (16) is:

(22) $$Z_{\nu }^{m,\alpha }\left(\rho \right)=\frac{\Gamma \left(m+1\right)}{\Gamma \left(m+1-\alpha \right)}\rho^{m-\alpha }+O\left(\rho^{m+2-\alpha }\right).$$

Since m−α>0, this expression tends to zero at the origin. When m=0, the constant term has zero Caputo derivative. The first potentially nonzero term is k=1; therefore,

(23) $$Z_{\nu }^{0,\alpha }\left(\rho \right)=A_{1}\left(\nu ,0\right)\frac{\Gamma \left(3\right)}{\Gamma \left(3-\alpha \right)}\rho^{2-\alpha }+O\left(\rho^{4-\alpha }\right),\;A_{1}\left(\nu ,0\right)=-\frac{\nu \left(\nu +2\right)}{4}.$$

Because 2−α>1, the feature is also bounded at the origin. Consequently, it admits the continuous extension $Z_{\nu }^{m,\alpha }\left(0\right)=0.$ At each interior point where the relevant limits are valid,

(24) $$\underset{\alpha \to 0^{+}}{lim}D_{0+}^{\alpha ,C}{\tilde{R}}_{\nu }^{m}\left(\rho \right)={\tilde{R}}_{\nu }^{m}\left(\rho \right)-{\tilde{R}}_{\nu }^{m}\left(0\right),$$

whereas

(25) $$\underset{\alpha \to 1^{-}}{lim}D_{0+}^{\alpha ,C}{\tilde{R}}_{\nu }^{m}\left(\rho \right)=\frac{d}{d\rho }{\tilde{R}}_{\nu }^{m}\left(\rho \right).$$

Therefore, the limit $\alpha \to 1^{-}$ recovers the ordinary radial derivative, not the underlying Zernike radial function itself. The classical Zernike polynomial is recovered through the integer-degree condition ν=m+2N, together with the normalization in Equation (15).

- Application to image-feature extraction

Let $I\left(\rho ,\theta \right)$ denote a grayscale image mapped to the normalized unit disk. A continuous generalized Zernike–Caputo feature coefficient may be defined as follows:

(26) $$M_{\nu ,m}^{\alpha }=\int_{0}^{2\pi }\int_{0}^{1-\varepsilon }I\left(\rho ,\theta \right)\bar{V_{\nu }^{m,\alpha }\left(\rho ,\theta \right)}\;\rho \;d\rho \;d\theta ,\;\;\varepsilon >0.$$

For a digital image, the corresponding discrete feature is:

(27) $$M_{\nu ,m}^{\alpha }=\sum_{i,j}I_{ij}\bar{V_{\nu }^{m,\alpha }\left(\rho_{ij},\theta_{ij}\right)}\;w_{ij},$$

where $w_{ij}$ denotes an optional quadrature or pixel-area weight. The use of 1−ε in (26) avoids the possible boundary growth of the nonterminating hypergeometric series. The parameter ν controls the generalized radial degree, m controls the azimuthal variation, and α is the Caputo differentiation order. These parameters have distinct mathematical roles.

- Data and Parameters Used in the 4×4 Example

Consider the grayscale image

(28) $$I=\left(\begin{array}{cccc} 0 & 1 & 2 & 3\\ 4 & 5 & 6 & 7\\ 8 & 9 & 10 & 11\\ 12 & 13 & 14 & 15 \end{array}\right).$$

The row and column indices are:

(29) i,j∈{0,1,2,3},

where i denotes the row index and j denotes the column index. The corresponding image intensity is:

(30) $$I_{ij}=4i+j.$$

The Cartesian coordinates associated with pixel $\left(i,j\right)$ are defined as follows:

(31) $$x_{ij}=j,\;\;y_{ij}=3-i,$$

so that the vertical coordinate increases upward. The geometric center of the image is:

(32) $$\left(x_{c},y_{c}\right)=\left(\frac{M-1}{2},\frac{N-1}{2}\right)=\left(\frac{3}{2},\frac{3}{2}\right),\;\;M=N=4.$$

The centered Cartesian coordinates are:

(33) $${\tilde{x}}_{ij}=x_{ij}-x_{c}=j-\frac{3}{2},\;\;{\tilde{y}}_{ij}=y_{ij}-y_{c}=\frac{3}{2}-i.$$

The unnormalized distance of pixel $\left(i,j\right)$ from the image center is:

(34) $$d_{ij}=\sqrt{{\tilde{x}}_{ij}^{\;2}+{\tilde{y}}_{ij}^{\;2}}=\sqrt{{\left(j-\frac{3}{2}\right)}^{2}+{\left(\frac{3}{2}-i\right)}^{2}}.$$

The largest center-to-corner distance is:

(35) $$d_{max}=\sqrt{{\left(\frac{3}{2}\right)}^{2}+{\left(\frac{3}{2}\right)}^{2}}=\sqrt{\frac{9}{2}}.$$

To keep all pixels strictly inside the unit disk, a small inward-scaling parameter is introduced as follows:

(36) $$\delta >0,\;\;\rho_{max}=\left(1+\delta \right)\sqrt{\frac{9}{2}}.$$

For example, one may use $\delta =10^{-2},$ where $d_{scale}=\left(1+\delta \right)d_{max}=\left(1+\delta \right)\sqrt{\frac{9}{2}},$ After applying the inward-scaling parameter δ>0, the normalized radii are defined by $\rho_{ij}=\frac{d_{ij}}{d_{scale}}.$ Consequently,

(37) ρmax:=max0≤i,j≤3ρij≈0.990099, δ=0.01,

where

$$\rho_{ij}=\frac{d_{ij}}{\rho_{max}}=\frac{\sqrt{{\left(j-\frac{3}{2}\right)}^{2}+{\left(\frac{3}{2}-i\right)}^{2}}}{\left(1+\delta \right)\sqrt{\frac{9}{2}}},\;\;0\leq \rho_{ij}<1.$$

The angular coordinate is determined by $atan2\left(y,x\right)$, which is the two-argument arctangent function,

(38) $$atan2\left(y,x\right)=\left\{\begin{array}{ll} arctan\left(\frac{y}{x}\right), & x>0,\\ arctan\left(\frac{y}{x}\right)+\pi , & x<0,\;y\geq 0,\\ arctan\left(\frac{y}{x}\right)-\pi , & x<0,\;y<0,\\ \frac{\pi }{2}, & x=0,\;y>0,\\ -\frac{\pi }{2}, & x=0,\;y<0, \end{array}\right.\;\;-\pi <atan2\left(y,x\right)\leq \pi ,$$

as follows:

(39) $$\theta_{ij}=atan2\left({\tilde{y}}_{ij},{\tilde{x}}_{ij}\right)=atan2\left(\frac{3}{2}-i,\;j-\frac{3}{2}\right),\;\;-\pi <\theta_{ij}\leq \pi .$$

The function $atan2\left(y,x\right)$ returns the polar angle of $\left(x,y\right)$ in the correct quadrant and remains defined when x=0 and y≠0. The function $atan2\left(y,x\right)$ returns the polar angle of $\left(x,y\right)$ in the correct quadrant and remains defined when x=0 and y≠0. For $\delta =10^{-2}$, the normalized radial-coordinate matrix is approximately:

(40) $$\rho_{approx}\approx \left(\begin{array}{cccc} 0.9901 & 0.7379 & 0.7379 & 0.9901\\ 0.7379 & 0.3300 & 0.3300 & 0.7379\\ 0.7379 & 0.3300 & 0.3300 & 0.7379\\ 0.9901 & 0.7379 & 0.7379 & 0.9901 \end{array}\right).$$

The angular-coordinate matrix is:

(41) $$\theta =\left(\begin{array}{cccc} \frac{3\pi }{4} & \pi -{tan}^{-1}\left(3\right) & {tan}^{-1}\left(3\right) & \frac{\pi }{4}\\ \pi -{tan}^{-1}\left(\frac{1}{3}\right) & \frac{3\pi }{4} & \frac{\pi }{4} & {tan}^{-1}\left(\frac{1}{3}\right)\\ -\pi +{tan}^{-1}\left(\frac{1}{3}\right) & -\frac{3\pi }{4} & -\frac{\pi }{4} & -{tan}^{-1}\left(\frac{1}{3}\right)\\ -\frac{3\pi }{4} & -\pi +{tan}^{-1}\left(3\right) & -{tan}^{-1}\left(3\right) & -\frac{\pi }{4} \end{array}\right).$$

The parameters of the generalized fractional-degree Zernike–Caputo feature are selected as $m\in Z,\;\nu \in R,\;\nu \geq \left|m\right|,\;0<\alpha <1,\;K\in N.$ For a specific illustration, one may take ν=1.5, m=0, α=0.35, K=10, δ=0.01. The coefficient of the truncated radial series is:

(42) $$A_{k}\left(\nu ,\left|m\right|\right)=\frac{{\left(\frac{\left|m\right|-\nu }{2}\right)}_{k}{\left(\frac{\left|m\right|+\nu }{2}+1\right)}_{k}}{{\left(\left|m\right|+1\right)}_{k}\;k!}.$$

The truncated Zernike–Caputo radial kernel is:

(43) $$Z_{\nu ,K}^{\left|m\right|,\alpha }\left(\rho_{ij}\right)=\sum_{\begin{array}{c} 0\leq k\leq K\\ \left|m\right|+2k>0 \end{array}}A_{k}\left(\nu ,\left|m\right|\right)\frac{\Gamma \left(\left|m\right|+2k+1\right)}{\Gamma \left(\left|m\right|+2k+1-\alpha \right)}\rho_{ij}^{\left|m\right|+2k-\alpha }.$$

Assuming equal pixel weights, $w_{ij}=1,\;i,j\in \{0,1,2,3\},$ the discrete generalized Zernike–Caputo feature is:

(44) Mν,m;Kα=∑i=03∑j=03Iij Zν,Km,αρije−imθij.

For the purely radial mode m=0, this reduces to:

(45) Mν,0;Kα=∑i=03∑j=03Iij Zν,K0,αρij,

For numerical computation, truncate the infinite series at k = K. The radial Caputo kernel is:

(46) $$Z_{\nu }^{m,\alpha }\left(\rho_{ij}\right)=\sum_{\begin{array}{c} k\geq 0\\ m+2k>0 \end{array}}\frac{{\left(\frac{m-\nu }{2}\right)}_{k}{\left(\frac{m+\nu }{2}+1\right)}_{k}}{{\left(m+1\right)}_{k}\;k!}\frac{\Gamma \left(m+2k+1\right)}{\Gamma \left(m+2k+1-\alpha \right)}\rho_{ij}^{m+2k-\alpha }.$$

Consequently, the practical form is:

(47) Mν,m;Kα=∑i=03∑j=03Iij∑0≤k≤Km+2k>0m−ν2km+ν2+1km+1k k!Γm+2k+1Γm+2k+1−αρijm+2k−αe−imθijwij.

- Projection onto the Fractional Zernike–Caputo Mode

The projection of the image I onto the generalized fractional-degree Zernike–Caputo mode is computed using the discrete inner product, as follows:

(48) Mν,m;Kα=I,Vν,m;Kαd=∑i=03∑j=03Iij Vν,m;Kαρij,θij¯,

where the overline denotes complex conjugation and

(49) $$V_{\nu ,m;K}^{\alpha }\left(\rho_{ij},\theta_{ij}\right)=Z_{\nu ,K}^{\left|m\right|,\alpha }\left(\rho_{ij}\right)e^{im\theta_{ij}}.$$

Therefore,

(50) Mν,m;Kα=∑i=03∑j=03Iij Zν,Km,αρije−imθij.

For the image $I=\left(\begin{array}{cccc} 0 & 1 & 2 & 3\\ 4 & 5 & 6 & 7\\ 8 & 9 & 10 & 11\\ 12 & 13 & 14 & 15 \end{array}\right),$ the projection can be written explicitly as follows:

(51) $$\begin{array}{c} M_{\nu ,m;K}^{\alpha }=\sum_{j=0}^{3}I_{0j}Z_{\nu ,K}^{\left|m\right|,\alpha }\left(\rho_{0j}\right)e^{-im\theta_{0j}}+\sum_{j=0}^{3}I_{1j}Z_{\nu ,K}^{\left|m\right|,\alpha }\left(\rho_{1j}\right)e^{-im\theta_{1j}}\\ +\sum_{j=0}^{3}I_{2j}Z_{\nu ,K}^{\left|m\right|,\alpha }\left(\rho_{2j}\right)e^{-im\theta_{2j}}+\sum_{j=0}^{3}I_{3j}Z_{\nu ,K}^{\left|m\right|,\alpha }\left(\rho_{3j}\right)e^{-im\theta_{3j}}. \end{array}$$

For the purely radial mode m=0, the angular factor satisfies $e^{-im\theta_{ij}}=1.$ Hence, the projection reduces to

(52) Mν,0;Kα=∑i=03∑j=03Iij Zν,K0,αρij.

Let

(53) $$K_{\nu ,0;K}^{\alpha }={\left(Z_{\nu ,K}^{0,\alpha }\left(\rho_{ij}\right)\right)}_{i,j=0}^{3}=\left(\begin{array}{cccc} K_{00} & K_{01} & K_{02} & K_{03}\\ K_{10} & K_{11} & K_{12} & K_{13}\\ K_{20} & K_{21} & K_{22} & K_{23}\\ K_{30} & K_{31} & K_{32} & K_{33} \end{array}\right)$$

denote the fractional radial kernel. Then, the discrete projection is the Frobenius inner product:

(54) Mν,0;Kα=I,Kν,0;KαF=∑i=03∑j=03IijKij.

For the specified image matrix, this becomes

(55) $$\begin{array}{c} M_{\nu ,0;K}^{\alpha }=0K_{00}+1K_{01}+2K_{02}+3K_{03}+4K_{10}+5K_{11}+6K_{12}+7K_{13}\\ +8K_{20}+9K_{21}+10K_{22}+11K_{23}+12K_{30}+13K_{31}+14K_{32}+15K_{33}. \end{array}$$

If the radial kernel is symmetric about the image center, then

(56) $$K_{00}=K_{03}=K_{30}=K_{33}=:K_{c},$$

(57) $$K_{01}=K_{02}=K_{10}=K_{13}=K_{20}=K_{23}=K_{31}=K_{32}=:K_{e},$$

and

(58) $$K_{11}=K_{12}=K_{21}=K_{22}=:K_{i}.$$

Consequently,

(59) Mν,0;Kα=30Kc+60Ke+30Ki.

The scalar $M_{\nu ,m;K}^{\alpha }$ measures the correlation between the image intensity distribution and the selected generalized fractional-degree Zernike–Caputo mode. A larger magnitude indicates stronger agreement between the spatial structure of the image and the corresponding radial-angular pattern. For the parameter values ν=1.5, m=0, α=0.35, K=10, δ=0.01, the fractional Zernike–Caputo kernel entries are defined as follows:

(60) $$K_{ij}=Z_{1.5,10}^{0,0.35}\left(\rho_{ij}\right)=\sum_{k=1}^{10}\frac{{\left(-\frac{3}{4}\right)}_{k}{\left(\frac{7}{4}\right)}_{k}}{{\left(k!\right)}^{2}}\frac{\Gamma \left(2k+1\right)}{\Gamma \left(2k+0.65\right)}\rho_{ij}^{2k-0.35}.$$

The normalized radial-coordinate matrix is:

(61) $$\rho =\left(\begin{array}{cccc} 0.990099 & 0.737976 & 0.737976 & 0.990099\\ 0.737976 & 0.330033 & 0.330033 & 0.737976\\ 0.737976 & 0.330033 & 0.330033 & 0.737976\\ 0.990099 & 0.737976 & 0.737976 & 0.990099 \end{array}\right),$$

where its approximation is as in Equation (40). Consequently, the kernel entries are:

(62) $$\begin{array}{rr} K_{00}=K_{03}=K_{30}=K_{33} & =Z_{1.5,10}^{0,0.35}\left(0.990099\right)\approx -2.976950,\\ K_{01}=K_{02}=K_{10}=K_{13}=K_{20}=K_{23}=K_{31}=K_{32} & =Z_{1.5,10}^{0,0.35}\left(0.737976\right)\approx -1.264517,\\ K_{11}=K_{12}=K_{21}=K_{22} & =Z_{1.5,10}^{0,0.35}\left(0.330033\right)\approx -0.290825. \end{array}$$

Therefore, the complete fractional Zernike–Caputo kernel matrix is:

(63) K1.5,0;100.35≈−2.976950−1.264517−1.264517−2.976950−1.264517−0.290825−0.290825−1.264517−1.264517−0.290825−0.290825−1.264517−2.976950−1.264517−1.264517−2.976950.

(64) M1.5,0;100.35≈−173.9043,  M1.5,0;100.35≈173.9043.

- Selection Criteria for the Fractional Parameters α and ν

The parameters α and ν have different mathematical and image-processing interpretations. The fractional degree ν controls the radial complexity of the generalized Zernike function, whereas the Caputo order α controls the strength of fractional differentiation. Therefore, they should be selected jointly rather than assigned the same interpretation. For an azimuthal order m, the parameters are restricted by $m\in N_{0},\;\nu \geq m,\;0<\alpha <1.$ The classical Zernike degrees are obtained when $\nu =m+2N,\;N\in N_{0}.$ A genuinely fractional-degree function is obtained when $\frac{\nu -m}{2}\notin N_{0}.$ For numerical image analysis, a bounded search region should be used: $\alpha \in \left[\alpha_{min},\alpha_{max}\right]\subset \left(0,1\right),\;\nu \in \left[m,\nu_{max}\right].$ A practical initial choice is $\alpha \in \left[0.1,0.9\right],\;\nu \in \left[m,m+6\right]$.

For interpretation of the Caputo order α, small values of α produce a weak fractional differentiation effect. In this case, the transformed radial kernel remains close to the underlying generalized Zernike function and primarily represents coarse intensity and shape information. Moderate values of α provide a balance between global radial structure and local texture variations. They are often suitable for biomedical images containing both smooth tissue regions and moderately irregular lesion textures. Large values of α make the operator closer to an ordinary first derivative. Consequently, they place greater emphasis on rapid intensity changes, edges, fine texture, and noise. Thus, the following categories apply:

(65) $$\begin{array}{cc} \mathrm{Range}\;\mathrm{of}\;\alpha & \mathrm{Dominant}\;\mathrm{image}\;\mathrm{information}\\ 0<\alpha <0.35 & \mathrm{coarse}\;\mathrm{structure}\;\mathrm{and}\;\mathrm{slowly}\;\mathrm{varying}\;\mathrm{intensity};\\ 0.35\leq \alpha \leq 0.7 & \mathrm{balanced}\;\mathrm{shape}–\mathrm{texture}\;\mathrm{information};\\ 0.7<\alpha <1 & \mathrm{edges},\;\mathrm{fine}\;\mathrm{texture},\;\mathrm{and}\;\mathrm{high}\text{-}\mathrm{frequency}\;\mathrm{variations}. \end{array}$$

The interval boundaries in (65) are practical guidelines rather than universal theoretical thresholds. The fractional degree ν controls the radial frequency and complexity of the kernel. Values of ν close to m generate smooth, slowly varying radial patterns. Increasing ν introduces more radial variation and allows the feature to represent smaller spatial structures. A practical interpretation is expressed as follows:

(66) $$\begin{array}{cc} \mathrm{Value}\;\mathrm{of}\;\nu & \mathrm{Dominant}\;\mathrm{radial}\;\mathrm{information}\\ \nu \approx m & \mathrm{coarse}\;\mathrm{radial}\;\mathrm{structure};\\ m<\nu ≲m+3 & \mathrm{intermediate}\;\mathrm{radial}\;\mathrm{texture};\\ \nu \gg m & \mathrm{fine}\;\mathrm{radial}\;\mathrm{details}\;\mathrm{and}\;\mathrm{possible}\;\mathrm{noise}\;\mathrm{sensitivity}. \end{array}$$

The upper bound $\nu_{max}$ should depend on the spatial resolution and the number of pixels in the region of interest. Very large values of ν should not be used for small images because the resulting radial variations cannot be adequately resolved.

- Resolution criterion

Let $N_{\rho }$ denote the effective number of radial samples in the normalized image region. The selected fractional degree should satisfy $\nu_{max}\ll 2N_{\rho },$ so that the radial kernel does not oscillate more rapidly than the available image resolution can represent. For a small 4×4 illustrative image, only low fractional degrees should be considered. A reasonable illustrative search set is $\nu \in \left\{m,\;m+0.5,\;m+1,\;m+1.5,\;m+2\right\}.$ For m=0, this becomes ν∈{0,0.5,1,1.5,2} The 4×4 matrix should be regarded only as a computational illustration. It is too small to reliably determine an optimal value for either parameter. For every candidate pair $\left(\alpha ,\nu \right)$, the truncated kernel should be numerically stable with respect to the truncation level K, defined as follows:

(67) $$E_{K}\left(\alpha ,\nu \right)=\underset{i,j}{max}\left|Z_{\nu ,K+1}^{m,\alpha }\left(\rho_{ij}\right)-Z_{\nu ,K}^{m,\alpha }\left(\rho_{ij}\right)\right|.$$

The truncation level is accepted when $E_{K}\left(\alpha ,\nu \right)<\varepsilon_{K},$ where $\varepsilon_{K}$ is a prescribed numerical tolerance, such as $10^{-6}$. Candidate parameter pairs that fail this condition should be removed from the search space. The extracted features should remain stable under small image perturbations, such as boundary shifts, intensity noise, resampling, or minor geometric transformations. Let $I^{\left(1\right)},\ldots ,I^{\left(B\right)}$ denote perturbed versions of the same image.

(68) $${\bar{M}}_{\alpha ,\nu }=\frac{1}{B}\sum_{b=1}^{B}M_{\alpha ,\nu }\left(I^{\left(b\right)}\right)$$

and

(69) $$S_{stab}\left(\alpha ,\nu \right)=\frac{\sqrt{\frac{1}{B-1}\sum_{b=1}^{B}{\left|M_{\alpha ,\nu }\left(I^{\left(b\right)}\right)-{\bar{M}}_{\alpha ,\nu }\right|}^{2}}}{\left|{\bar{M}}_{\alpha ,\nu }\right|+\epsilon },$$

where ϵ>0 prevents division by zero. Smaller values of $S_{stab}$ indicate greater stability. Alternatively, feature reproducibility may be assessed using the intraclass correlation coefficient. Candidate parameters (ICC) may be required to satisfy $ICC\left(\alpha ,\nu \right)\geq 0.75,$ with ICC≥0.90 indicating excellent stability. For a classification problem, the optimal parameters should be selected using only the training data. Let ${AUC}_{CV}\left(\alpha ,\nu \right)$ denote the cross-validated area under the receiver-operating-characteristic curve. The performance-based parameter pair is:

(70) $$\left(\alpha^{*},\nu^{*}\right)=\underset{\left(\alpha ,\nu \right)\in \Omega }{arg\;max}\;{AUC}_{CV}\left(\alpha ,\nu \right),$$

where Ω is the admissible and numerically stable parameter region. For imbalanced data, balanced accuracy, the $F_{1}$ score, or the area under the precision–recall curve may be used instead of the ordinary accuracy. A more reliable procedure combines predictive performance, feature stability, and model complexity.

(71) $$J\left(\alpha ,\nu \right)=L_{CV}\left(\alpha ,\nu \right)+\lambda_{s}S_{stab}\left(\alpha ,\nu \right)+\lambda_{\nu }{\left(\frac{\nu -m}{\nu_{max}-m}\right)}^{2},$$

where:

- $L_{CV}$ is the cross-validated prediction loss;
- $S_{stab}$ is the perturbation-stability penalty;
- the last term penalizes unnecessarily large fractional degrees;
- $\lambda_{s}\geq 0$ and $\lambda_{\nu }\geq 0$ are regularization parameters.

The selected parameters are

(72) α∗,ν∗=arg minα,ν∈Ω Jα,ν.

A practical candidate grid is α∈{0.10,0.20,…,0.90}, and

ν∈{m,m+0.5,m+1,…,m+6}.

The selection procedure consists of the following steps:

- Generate the candidate pairs $\left(\alpha ,\nu \right)$ from the search grid;
- Remove parameter pairs that fail the convergence condition;
- Extract the generalized Zernike–Caputo features for the remaining parameter pairs;
- Evaluate stability under segmentation, intensity, and resampling perturbations;
- Evaluate predictive performance using nested cross-validation;
- Select the pair minimizing (71);
- Refit the final feature-extraction and classification pipeline on the complete training set;
- Evaluate the selected pair only once on the independent test set.

**Figure 4:**
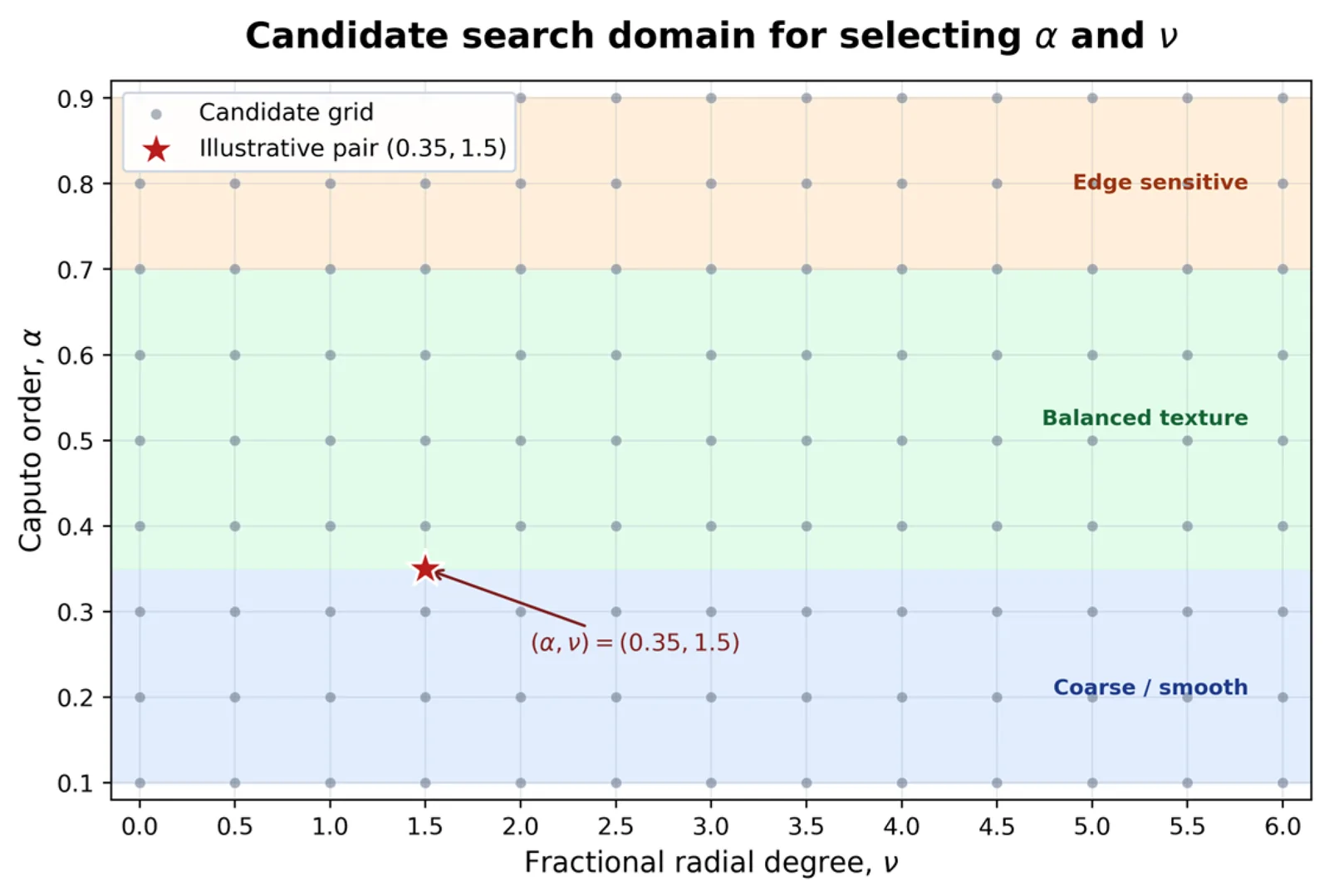
Candidate search domain for the Caputo order α and fractional radial degree ν.

Nested cross-validation is important because selecting $\left(\alpha ,\nu \right)$ and reporting performance on the same data may produce an optimistically biased estimate. For the 4×4 numerical example, the values α=0.35, ν=1.5 represent a moderate fractional differentiation order and an intermediate fractional radial degree. These values are suitable for demonstrating the computation, but they should not be described as universally optimal. For a real image dataset, the final values of α and ν must be estimated from the training data using the convergence, stability, and cross-validation criteria described above (see Figure 4). The pseudocode of the proposed Fractional Zernike–Caputo Features used for handcrafted feature extraction from liver DCE-MRI images is illustrated in Algorithm 1.

The shaded regions indicate the approximate image characteristics emphasized by different values of α. The pair $\left(\alpha ,\nu \right)=\left(0.35,1.5\right)$ is used only for numerical illustration; final parameter selection should be based on convergence, feature stability, and nested cross-validation.
**Algorithm 1.** Pseudocode of the proposed fractional Zernike–Caputo features.Begin For each fractional degree α ∈ A do  Initialize feature set F(α) ← ∅  For each image I ∈ D do    **Step 1: Preprocessing**    Convert I to grayscale    Remove black border from I    Crop I to a square shape     **Step 2: Fractional Zernike Feature Extraction**    Convert image coordinates to polar form (r, θ)    Set fractional degree parameter to α    For order p = 0 to p_max do     Compute fractional-degree Zernike radial polynomial     Extract corresponding fractional Zernike moments     End For     **Step 3: Feature Reduction**     Compute mean of Zernike moments     Compute standard deviation of Zernike moments     Compute skewness of Zernike moments     Compute kurtosis of Zernike moments     Concatenate reduced statistics into feature vector f     Add f to F(α)     End For     **Step 4: Save Features**     Store feature set F(α)  End ForEnd

D. Feature Processing and Integration

The proposed framework integrates heterogeneous features extracted from time–intensity curves (TICs), deep convolutional neural networks (CNNs), and fractional Zernike–Caputo (FZC) descriptors through a structured feature fusion pipeline. The pipeline consists of the following steps:

1. Feature Normalization

Each feature set is independently normalized using z-score normalization:

$$\hat{x}=\frac{x-\mu }{\sigma },$$

where μ  and σ denote the mean and standard deviation computed from the training data only. This standardization ensures that no feature domain dominates the classification process because of differences in scale.

2. Feature Selection

To eliminate redundant and highly correlated variables while preserving the most discriminative information, a feature selection stage has been introduced prior to fusion. This step reduces feature redundancy, decreases computational complexity, and improves classifier generalization.

3. Feature Fusion Strategy

To explain that the proposed framework employs an early feature-level fusion strategy. After normalization and feature selection, the three complementary feature vectors are concatenated into a single hybrid representation:

$$F_{Hybrid}=[F_{TIC},F_{CNN},F_{FZC}]$$

where $F_{TIC}$ represents the temporal physiological characteristics extracted from the dynamic time–intensity curves, F_CNN contains high-level semantic image features learned by the convolutional neural network, and $F_{FZC}$ consists of the proposed fractional Zernike–Caputo descriptors encoding fractional geometric and shape information. This fusion strategy preserves the complementary information contained in each feature domain while producing a unified representation for subsequent classification.

### 2.4. Classifier Development

Several machine-learning classifiers, including support vector machine (SVM), random forest (RF), and extreme gradient boosting (XGBoost), were evaluated using the same cross-validation framework and input features. All data-dependent preprocessing and model-development procedures were performed using the training data within each cross-validation iteration, while the corresponding held-out fold was reserved exclusively for final performance evaluation. For the SVM classifier, a Gaussian radial basis function (RBF) kernel was used with a box constraint of 1 and a kernel scale of 2.4. Feature standardization was performed using parameters derived from the training data and subsequently applied unchanged to the corresponding held-out fold. RF and XGBoost classifiers were implemented in MATLAB and evaluated using the same cross-validation procedure. The performance of all classifiers was subsequently compared using the same evaluation criteria. Among the evaluated classifier configurations, SVM achieved the highest classification performance.

## 3. Experimental Results

The experimental results reveal that the best performance of classifying liver DCE-MRI into benign and malignant cancer was achieved when all the extracted features were combined. In this study, two sets of feature vectors were extracted from each time phase of the liver DCE-MRI scans (t_0_, t_1_, t_2_, and t_3_), and two and four features per phase were extracted by CNN and fractional Zernike–Caputo methods, respectively. resulting in eight deep features and 16 fractional Zernike–Caputo features across four phases, respectively. In addition, seven kinetic features were extracted from the TICs. Therefore, the complete fused feature vector included 31 features extracted from four-time phases of each liver DCE-MRI sequence.

MATLAB 2025a was used to implement the proposed algorithm. The performance of the proposed method was evaluated using five-fold cross-validation. The dataset comprising 645 patients was randomly divided into five approximately equal subsets. In each iteration, four subsets were used for model training and the remaining subset was retained as an unseen testing fold. This procedure was repeated five times so that each subset was used once as the testing fold. Thus, five classification results were obtained using different training and testing subsets, and the final performance was calculated by averaging the results across the five folds. For each cross-validation iteration, the feature-processing and classification procedures were applied using the corresponding training and testing subsets. Feature normalization parameters were calculated from the training data and subsequently applied to the corresponding testing data. The SVM classifier was trained using the training-fold features and evaluated using the held-out testing fold. The performance measures obtained from the five folds were then aggregated and reported as the mean ± standard deviation (SD).

To prevent information leakage, all data-dependent preprocessing and model-development operations were performed independently within each cross-validation iteration. Specifically, feature normalization parameters (mean and standard deviation) were estimated exclusively from the training folds and subsequently applied unchanged to the corresponding held-out fold. Feature selection was also performed using the training folds only, and the selected feature subset was then applied to the held-out fold without recalculation. Similarly, SVM hyperparameters were determined using only the training data. The held-out fold remained completely unseen during preprocessing, feature selection, parameter optimization, and model training and was used only for final performance evaluation.

This study assessed the effectiveness of the proposed CNN that was used for deep-feature extraction from the cropped pre-contrast and multi-phase post contrast images (first, second, and third phases). Such images in DCE-MRI sequences are particularly essential for diagnosing and classifying liver lesions into benign and malignant. From both the early and late post-contrast phases, eight deep features were extracted by CNN. The proposed CNN was trained using the Adam optimizer with an initial learning rate of 10^−4^, a maximum of 50 epochs, and shuffling at every epoch. The network was initialized using MATLAB’s default initialization for the layers. A fixed random seed (672880951) was used to improve reproducibility. The CNN architecture consisted of convolutional layers with 64, 128, 256, and 128 filters, respectively, followed by fully connected layer, as described in Table 1. The network contained 786,574 trainable parameters. Data augmentation was applied using random horizontal reflection, random rotation between −20° and +20°, and random scaling between 0.8 and 1.2. The input images were resized/preprocessed to 141 × 113 × 1.

**Figure 5:**
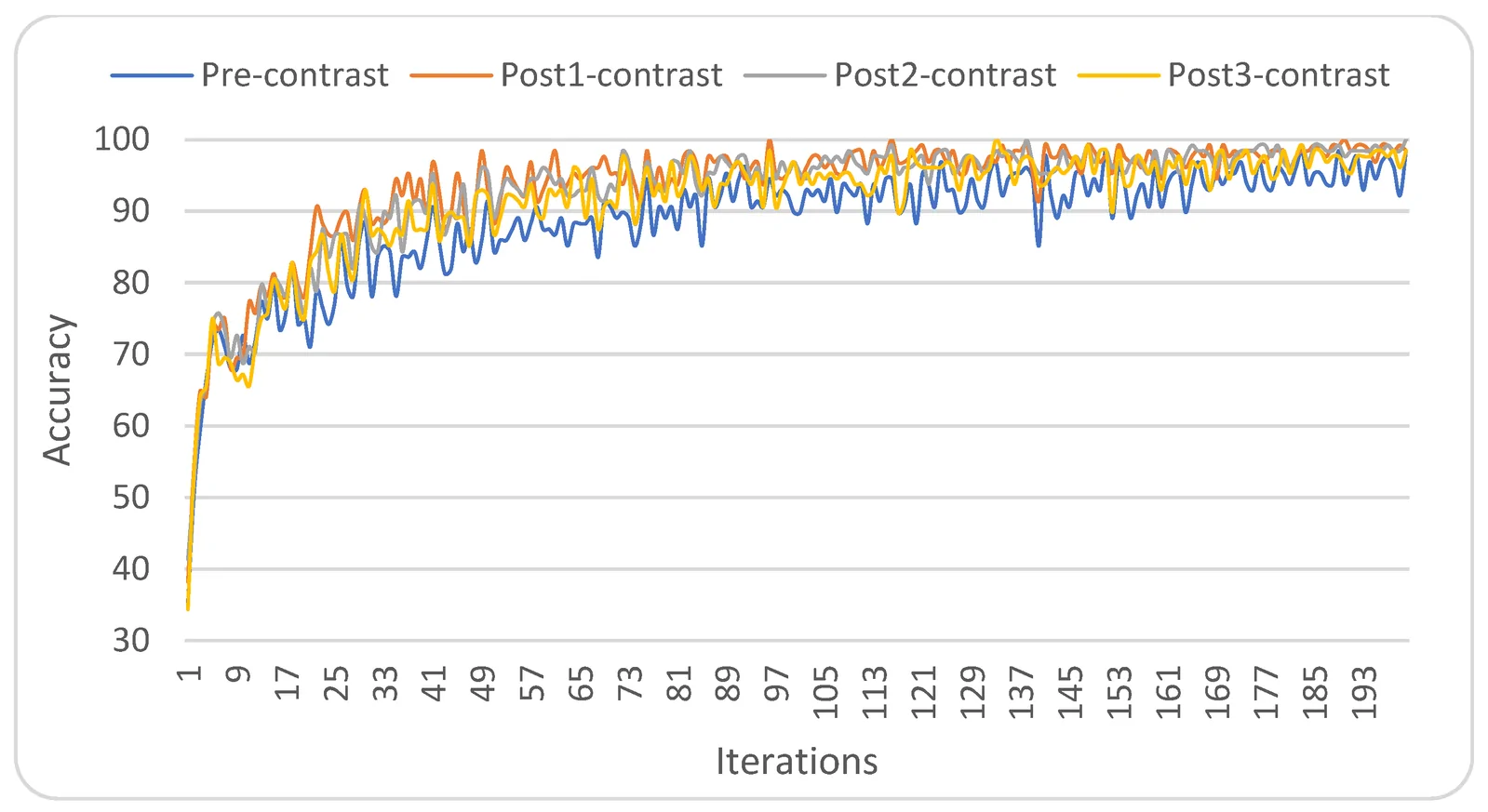
The proposed CNN model’s pre-contrast and post-contrast training plots at t_1_, t_2_, and t_3_, respectively.

**Figure 6:**
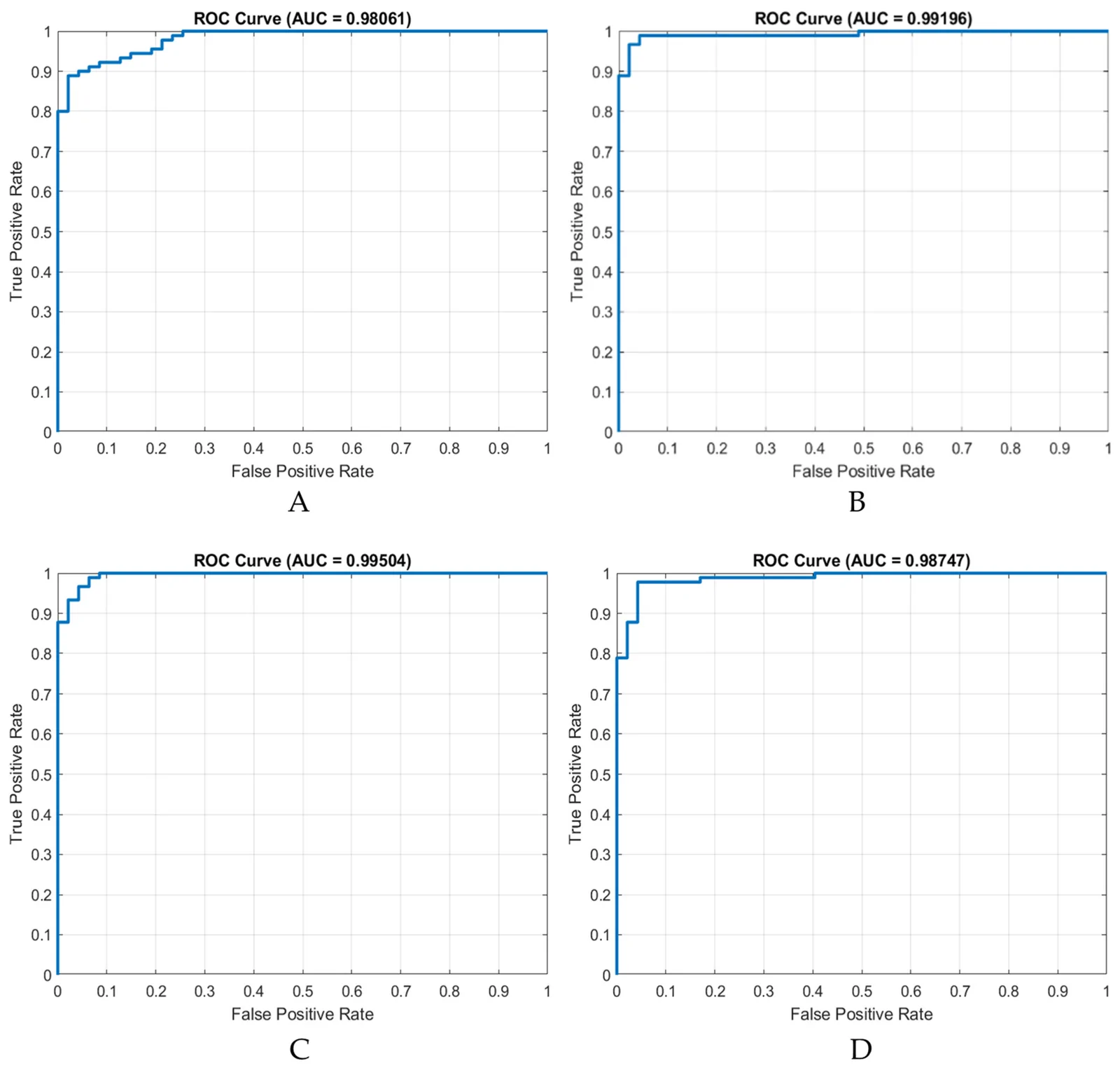
ROC curves showing the performance of the CNN model during evaluation of the deep-feature extraction network: (**A**) pre-contrast phase, (**B**) post-1 contrast phase, (**C**) post-2 contrast phase, and (**D**) post-3 contrast phase.

Figure 5 and Figure 6 show the evolution of training accuracy and the receiver operating characteristic (ROC) curves for the test data, respectively, demonstrating the performance of the proposed CNN model in extracting deep features (DF) from the single pre-contrast and three successive post-contrast MRI dynamics.

The performance of the proposed model in distinguishing between benign and malignant liver lesions from DCE-MRI scans was completely evaluated and compared through utilizing a set of metrics (accuracy, area under the curve (AUC), sensitivity, and specificity), all derived from the initial four fundamental counts: true positive (TP), true negative (TN), false positive (FP), and false negative (FN) [26,33,34]. The experiment results indicate that the post-contrast features provided substantially better diagnostic performance than pre-contrast or TIC features, in all measured metrics of both the post-1 and post-2 contrast phases, while the weakest overall accuracy and AUC were achieved by TIC, at 87.30% and 90.58%, respectively.

**Figure 7:**
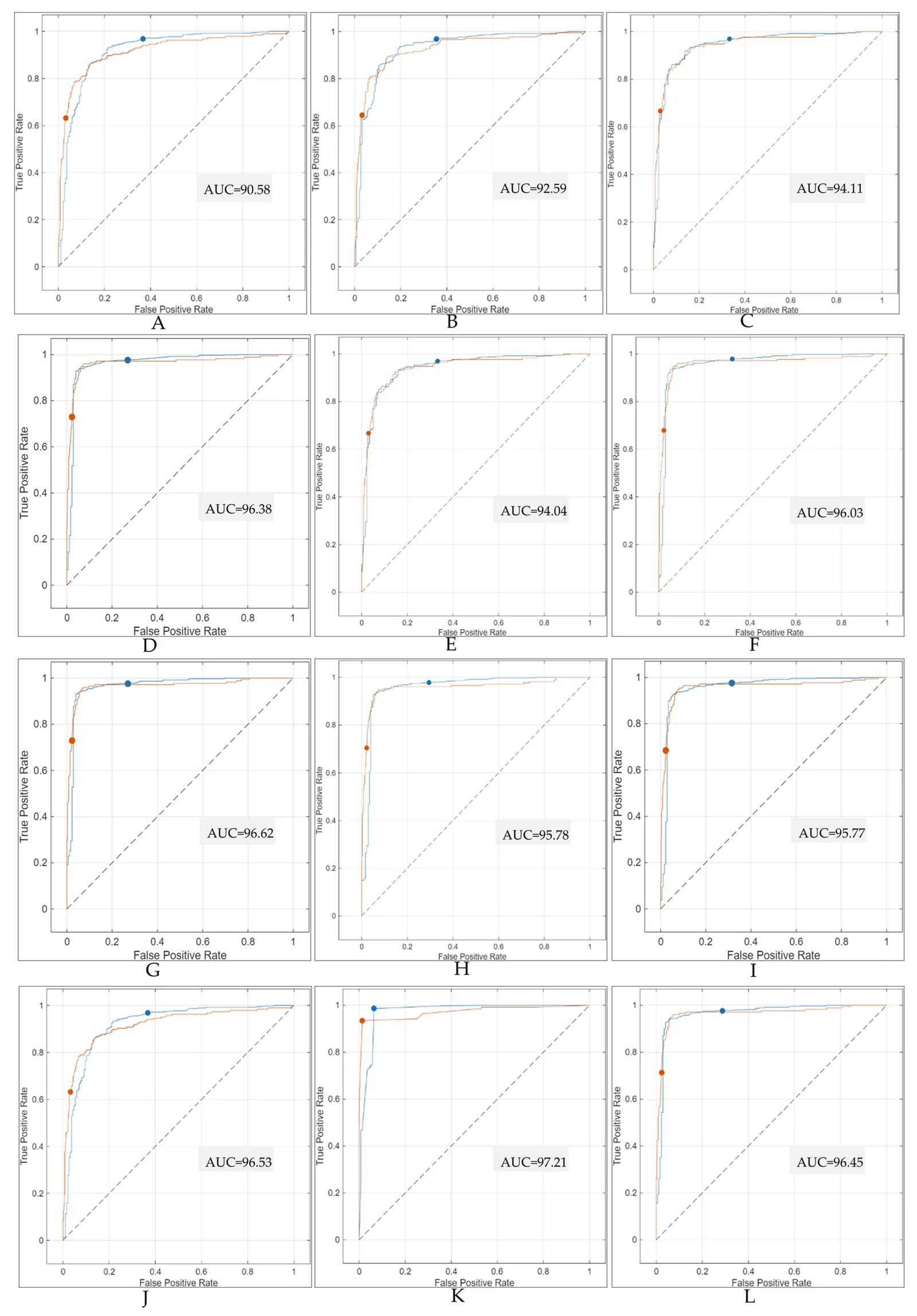

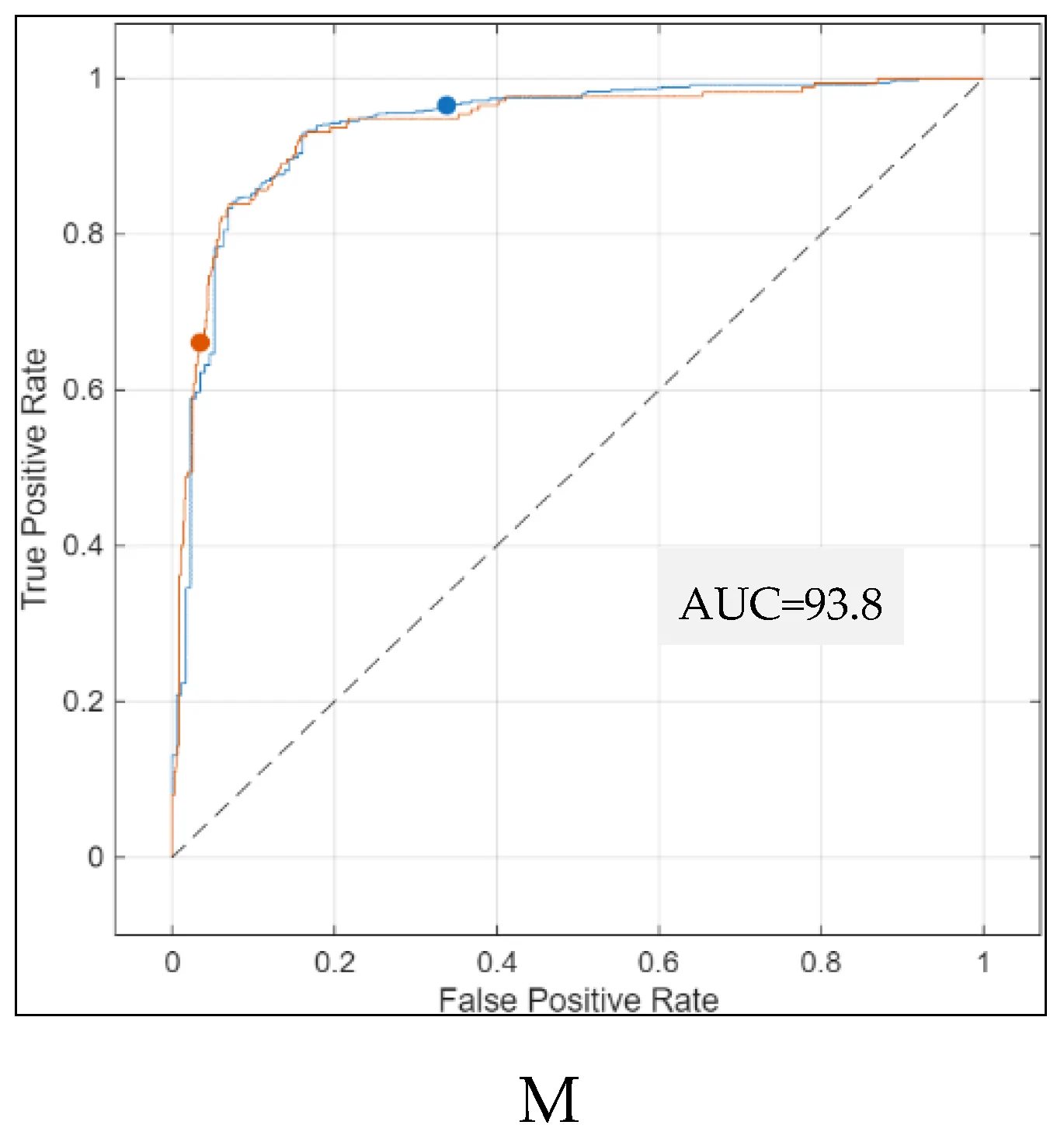
ROC curves for the classification performance (blue and orange curves are for benign and malignant lesions respectively), (**A**) Kinetic features, (**B**) FZ Pre-contrast, (**C**) FZ Post-contrast 1, (**D**) FZ Post-contrast 2, (**E**) FZ Post-contrast 3, (**F**) DF Pre-contrast, (**G**) DF Post-contrast 1, (**H**) DF Post-contrast 2, (**I**) DF Post-contrast 3, (**J**) Combined Features of Pre-contrast, (**K**) Combined Features of Post 1-contrast, (**L**) Combined Features of Post 2-contrast, (**M**) Combined Features of Post 3-contrast.

This indicates that utilizing dynamic curves individually is less discriminative. While both FZ and DF features showed improvements after contrast injection, FZ post2 features achieved one of the highest single-feature performances with accuracy and AUC values of 92.82% and 96.38%, respectively. The diagnosis performance yielded further gains when FZ and DF features were combined, especially in the post-1 phase, which recorded the best overall performance with accuracy, sensitivity, specificity, and AUC values of 94.05%, 94.17%, 93.95%, and 97.21%, respectively, as shown in Figure 7. Additionally, the combined FZ and DF features strongly improved the performance of the pre-contrast phase to yield an accuracy of 92.53%, with perfect sensitivity and TN values. However, the post-contrast combination of FZ and DF features significantly outperformed all other individual features, particularly in the post-1 and post-2 phases, achieving AUCs of 97.21% and 96.45%, respectively, as shown in Table 2.

Although the combined post-contrast phases yield the highest overall diagnostic performance, the combined pre-contrast information is associated with remarkably strong results and may be sufficient to distinguish between benign and malignant cancer. This indicates that relying on pre-contrast information can reduce patient exposure to contrast material, which is particularly beneficial for patients who suffer from allergies, impaired renal function, or other contraindications. Furthermore, avoiding the use of contrast material can be cost-effective and reduce scan time [29,35].

To prevent information leakage and obtain an unbiased estimate of the model performance, a nested patient-level cross-validation strategy was employed. The dataset was divided into outer folds at the patient level, ensuring that all images/lesions belonging to the same patient were assigned exclusively to either the training or test partition within each fold. For each outer fold, the CNN was trained from scratch using only the patients included in the corresponding outer training set. Deep features were subsequently extracted using the CNN trained within that fold, and no images from the outer test set were used during CNN training or feature extraction model development.

Within each outer training set, model-development procedures and SVM hyperparameter optimization were performed using inner cross-validation. Feature preprocessing, feature selection, and SVM parameter optimization were restricted to the inner training data and subsequently applied to the corresponding validation data. The final SVM model for each outer fold was then trained using the complete outer training set with the selected parameters and evaluated once on the completely unseen outer test patients. The performance values reported in this study represent the aggregate performance across the outer test folds.

The extracted features were classified using a support vector machine (SVM) with a Gaussian kernel. The kernel scale was set to 2.4, and the box constraint (C) was set to 1. All features were standardized using parameters estimated from the training data only. To prevent information leakage, all SVM model-development procedures were performed exclusively within the training portion of each outer patient-level cross-validation fold. Data for the corresponding outer test patients were kept completely unseen during feature preprocessing and SVM model development and were used only for final evaluation. Classification performance was determined from the predictions on the outer test folds and summarized across the outer folds. Table 3 presents the mean accuracy and standard deviation obtained across the outer test folds for all feature configurations. The combined post-1-contrast features achieved the highest mean accuracy with relatively low variability across the outer folds.

For further evaluation to select the effectiveness of SVM classifier, an additional experiment was conducted to compare the performance of SVM classifier with three alternative classifiers: Random Forest, XGBoost, and end-to-end CNN classification. The SVM achieved the highest classification accuracy (94.05%) among the evaluated classifiers, followed by Random Forest (93.02%), end-to-end CNN (92.84%), and XGBoost (92.66%), as demonstrated in Table 4.

To verify the effectiveness of the proposed method, several previous studies on liver DCE-MRI diagnosis are summarized in Table 5, based on the used image modality, feature extraction strategy, dataset size, and classifier. Across the reviewed studies [21,22,23], CT based liver diagnosis generally depicts lower performance compared to MRI due to limitation of functional contrast in liver diagnosis especially for small or early-stage lesions [36]. Classical and extensive radiomic approaches achieved moderate accuracies between 80% to 92.68%. On the other hand, the DCE-MRI based deep learning approaches yielded high discriminative power due to capturing dynamic vascular behavior, which is an important feature to differentiate between benign and malignant liver cancers. For example, Du, Yuan [24], reported the AUC values using SVM and DenseNet individually were 85.7% and 83.5%, respectively. In contrast, combining handcrafted radiomic and deep-learning features improved the discrimination power of the classifier, as demonstrated by Wu, Zhang [25], who achieved an accuracy and AUC of 93.5% and 98.8%, respectively. Fotouhi, Samadi Khoshe Mehr [37] used visual LI-RADS-based features with SVM to achieve an accuracy and AUC of 82% and 80%, respectively. Another study utilized a DCE-MRI-based deep-learning approach and yielded an accuracy of approximately 94%. In the present study, the integration of kinetic, fractional Zernike–Caputo (FZC), and deep-learning features achieved an accuracy of 94.05% and an AUC of 97.21% at the post-1 phase. The relatively large cohort of 645 liver DCE-MRI cases represents an important strength of the study and provides a substantial dataset for model development and evaluation. However, cohort size alone does not necessarily determine diagnostic performance. The performance values reported in Table 5 should therefore be interpreted with caution because the included studies differed in imaging modalities, patient populations, sample sizes, lesion categories, classification objectives, feature extraction strategies, reference standards, and validation protocols. Some studies focused on specific tumor subtypes or multi-class classification tasks, whereas the present study focused on differentiating benign from malignant focal liver lesions. Differences in DCE-MRI acquisition protocols, dataset composition, and training and testing procedures may also have influenced the reported diagnostic performance. Therefore, the accuracy and AUC values in Table 5 should not be considered direct head-to-head comparisons or evidence of superiority. Rather, Table 5 provides a contextual overview of the methodological approaches and reported performance in the existing literature. Within this context, the main methodological contribution of the present study is the fusion of complementary kinetic, FZC, and deep features, which provides a more comprehensive representation of focal liver lesions.

## 4. Ablation Study

In the ablation study, we investigated the contribution of utilizing TIC, FZ, DF features and contrast phases of liver MRI cancers. The post-contrast phases, especially post-1 and post-2, provided the highest diagnostic performance across all phases. The combined post-1 achieved the overall best results for accuracy, sensitivity, specificity, and AUC at 94.05%, 94.17%, 93.95% and 97.21%, respectively. This was followed closely by FZ post-2 and DF post-2, both yielding AUC values above 95%. Despite the clear advantages of utilizing contrast enhancement, the combined pre-contrast features also showed significant performance, achieving accuracy and AUC values of 92.53% and 96.53%, respectively. Because most discriminative information was already presented in the pre-contrast MRI live sequences, the combined FZ and DF features’ performance outperformed the single pre-contrast phases and dramatically surpassed that of the TIC features, which achieved the weakest performance. Further another ablation studies were performed to choose the optimal structure of the proposed CNN to extract the best DF in terms of its contribution to diagnostic performance across DCE-MRI phases, implementing nine experiments that included altering the numbers of convolutional layers and kernel filters in each experiment to evaluate the performance of the extracted DF features, as illustrated in Table 6.

The present study evaluated the use of deep CNN-derived features and conventional texture features for the classification of focal liver lesions from DCE-MRI. The results demonstrated differences in classification performance among the evaluated feature configurations, with the combined post-contrast features providing the most favorable overall performance. This finding suggests that complementary information from deep and texture-based representations can contribute to a more informative characterization of focal liver lesions compared with either representation alone. The improved performance obtained using the combined features may be explained by the complementary characteristics of the two feature types. CNN-derived deep features provide hierarchical representations of image appearance and can capture complex spatial patterns that may not be explicitly represented by conventional handcrafted descriptors. In contrast, texture features quantify local intensity relationships and tissue heterogeneity. Combining these representations may therefore allow the classifier to exploit both higher-level spatial information and local textural characteristics of the lesions. The contribution of post-contrast information is also clinically plausible because contrast enhancement reflects differences in lesion vascularity and enhancement behavior, which are important characteristics in the radiological assessment of focal liver lesions.

The performance of the SVM classifier further supports the usefulness of the extracted feature representations. A Gaussian-kernel SVM was used to model potentially nonlinear relationships between the extracted features. This approach is particularly suitable for feature-based classification when the feature dimensionality is relatively high compared with the number of patients. The relatively consistent performance across the evaluation folds also indicates that the classification results were not dependent on a single subset of patients.

An important strength of the revised analysis is the use of patient-level validation to reduce the possibility of information leakage. In particular, patients were separated at the outer validation level so that information from the same patient was not simultaneously available for model development and final evaluation. CNN training and deep-feature extraction were performed using the corresponding training patients, while data for the outer test patients remained unseen until evaluation. Similarly, feature preprocessing and SVM model development were restricted to the training data. This procedure provides a more rigorous estimate of the generalization performance of the proposed approach than an evaluation in which images from the same patient may be present in both training and test data.

The findings indicate that the proposed feature-based framework may provide a useful computer-aided approach for analyzing focal liver lesions in DCE-MRI. Rather than relying exclusively on either deep learning representations or conventional texture descriptors, the results support the potential value of integrating complementary image information within a conventional supervised classifier. Nevertheless, the observed performance should be considered an experimental result requiring further validation before clinical implementation. The main limitations of the study and directions for future work are presented in the Conclusions section.

## 5. Conclusions

This study presents a hybrid, automated study for classifying liver masses into benign and malignant categories using dynamic contrast-enhanced MRI (DCE-MRI). The approach integrates three complementary feature types, namely, time–intensity curve (TIC) characteristics, deep learning features, and fractional Zernike–Caputo descriptors, to capture kinetic behavior, complex spatial patterns, and advanced texture information, respectively. By fusing these heterogeneous features into a unified representation, the proposed model achieved a high diagnostic accuracy of 94.05% on a dataset of 645 dynamic MRI cases. The results demonstrate the effectiveness of combining kinetic, data-driven, and fractional mathematical descriptors for improving liver lesion characterization. This study has strong potential to assist clinicians in making more reliable, non-invasive diagnostic decisions, thereby reducing dependence on biopsy procedures and supporting the evaluation of ambiguous cases. However, this study has several limitations. First, the dataset was collected from a single imaging center using a 1.5-T Philips Achieva MRI system, which may limit the generalizability of the proposed model to other institutions, MRI vendors, field strengths, acquisition protocols, and patient populations. Although five-fold cross-validation was performed, this represents internal validation and is not a substitute for independent external validation. Future work should therefore evaluate the locked model on independent multicenter cohorts acquired using different MRI systems and acquisition protocols. Such validation should include standardized preprocessing procedures, center-specific and pooled assessments of AUC, accuracy, sensitivity, specificity, confidence intervals, and calibration, together with analysis of feature stability and reproducibility across centers. Appropriate harmonization strategies, including standardized intensity normalization and statistical harmonization methods such as ComBat, should also be investigated to reduce potential site- and scanner-related variability. Following retrospective multicenter external validation, prospective evaluation across multiple institutions should be conducted to determine the robustness and clinical utility of the proposed framework.

## Figures and Tables

**Table 1:** The proposed architecture of CNN.

Layer Name	Kernel Filter	Kernel Size	Feature Map	Parameters
Input layer		(113 × 141 × 1)		
Convolution layer 1	64	(3 × 3)	(113 × 141 × 64)	(3 × 3 × 1 × 64) + 64 = 640
Pooling layer 1		(2 × 2)	(56 × 70 × 64)	
Convolution layer 2	128	(3 × 3)	(56 × 70 × 128)	(3 × 3 × 64 × 128) + 128 = 73,856
Pooling layer 2		(2 × 2)	(28 × 35 × 128)	
Convolution layer 3	256	(3 × 3)	(28 × 35 × 256)	(3 × 3 × 128 × 256) + 256 = 295,168
Convolution layer 4	128	(3 × 3)	(28 × 35 × 128)	(3 × 3 × 256 × 128) + 128 = 295,040
Pooling layer 3		(2 × 2)	(14 × 17 × 128)	
Fully connected layer 1		(1 × 4)	(1 × 4)	(14 × 17 × 128 × 4) + 4 = 121,860
Fully connected layer 2		(1 × 2)	(1 × 2)	(4 × 2) + 2 = 10
Total				786,574

**Table 2:** Achieved results by combining the proposed FZ with kinetic and DF features using an SVM classifier.

Features	ACC (%)	Sensitivity (%)	Specificity (%)	AUC (%)
TIC features	87.30	89.30	86.54	90.58
FZ Pre-contrast	89.10	87.32	89.83	92.59
FZ Post-1 contrast	91.96	92.85	91.50	94.11
FZ Post-2 contrast	92.82	91.37	93.52	96.38
FZ Post-3contrast	91.30	90.43	92.68	94.04
DF Pre-contrast	92.06	91.30	92.48	96.03
DF Post-1 contrast	92.82	92.91	92.71	96.62
DF Post-2 contrast	92.63	91.36	93.31	95.78
DF Post-3 contrast	92.34	89.18	94.14	95.77
TIC + FZ Pre-contrast	91.10	90.25	91.72	95.05
TIC + FZ Post-1 contrast	93.05	92.68	93.41	96.35
TIC + FZ Post-2 contrast	92.25	90.92	93.38	96.02
TIC + FZ Post-3 contrast	90.05	88.82	91.12	93.20
TIC + DF Pre-contrast	92.00	92.00	92.00	92.00
TIC + DF Post-1 contrast	93.40	93.40	93.40	93.40
TIC + DF Post-2 contrast	92.40	92.40	92.40	92.40
TIC + DF Post-3 contrast	90.20	90.20	90.20	90.20
FZ + DF Pre-contrast	92.20	92.20	92.20	92.20
FZ + DF Post-1 contrast	93.65	93.65	93.65	93.65
FZ + DF Post-2 contrast	92.55	92.55	92.55	92.55
FZ + DF Post-3 contrast	90.35	90.35	90.35	90.35
Combined Pre-contrast	92.53	95.00	91.35	96.53
Combined Post-1 contrast	94.05	94.17	93.95	97.21
Combined Post-2 contrast	92.63	91.00	93.51	96.45
Combined Post-3 contrast	90.44	89.19	91.04	93.80

**Table 3:** Classification accuracy across five folds of cross-validation for the different feature configurations. Values are presented as accuracy (%) for each fold and as mean ± standard deviation (SD).

Features	Fold 1	Fold 2	Fold 3	Fold 4	Fold 5	Mean ± SD
TIC features	85.40	88.90	87.80	86.50	87.90	87.30 ± 1.42
FZ Pre-contrast	87.30	90.20	89.80	88.10	90.10	89.10 ± 1.26
FZ Post-1 contrast	90.30	92.80	91.40	92.70	92.60	91.96 ± 1.08
FZ Post-2 contrast	91.50	93.80	92.40	93.20	93.20	92.82 ± 0.93
FZ Post-3 contrast	89.60	92.30	91.80	91.10	91.70	91.30 ± 1.01
DF Pre-contrast	90.50	93.10	92.70	91.20	92.80	92.06 ± 1.09
DF Post-1 contrast	91.30	94.10	92.50	93.40	92.80	92.82 ± 1.07
DF Post-2 contrast	91.30	93.70	92.40	93.00	92.80	92.64 ± 0.92
DF Post-3 contrast	91.00	93.70	92.80	91.30	92.90	92.34 ± 1.13
TIC + FZ Pre-contrast	90.20	92.40	91.60	91.80	91.70	91.54 ± 0.87
TIC + FZ Post-1 contrast	92.30	93.80	93.10	93.50	93.20	93.18 ± 0.57
TIC + FZ Post-2 contrast	91.70	93.20	92.20	92.80	92.50	92.48 ± 0.59
TIC + FZ Post-3 contrast	89.70	91.30	90.50	90.80	90.60	90.58 ± 0.62
TIC + DF Pre-contrast	91.50	93.40	92.20	92.80	92.60	92.50 ± 0.74
TIC + DF Post-1 contrast	92.70	94.10	93.40	93.70	93.50	93.48 ± 0.54
TIC + DF Post-2 contrast	91.80	93.40	92.40	92.90	92.60	92.62 ± 0.61
TIC + DF Post-3 contrast	89.80	91.50	90.60	90.90	90.70	90.70 ± 0.67
Combined Pre-contrast	91.30	93.70	92.80	92.00	92.90	92.54 ± 0.96
Combined Post-1 contrast	93.20	94.80	94.10	93.70	94.40	94.05 ± 0.62
Combined Post-2 contrast	91.30	93.70	92.40	93.00	92.80	92.64 ± 0.92
Combined Post-3 contrast	89.00	91.70	90.20	91.00	90.30	90.44 ± 1.10

**Table 4:** Comparison of performance for several classifiers with the achieved accuracy of other classifiers.

Classifier	Accuracy (%)	Sensitivity (%)	Specificity (%)
XGBoost	92.66	91.05	94.04
Random Forest	93.02	91.43	94.37
End-to-end CNN	92.84	91.24	94.20
SVM	94.05	93.97	94.57

**Table 5:** Comparisons between the proposed model and alternative techniques for liver DCE-MRI diagnosis.

Refs.	Dataset	Features	Classifier	Accuracy 100%	AUC 100%
[21]	601 CT scans (208 benign, 200 malignant, 193 metastasis)	- Histogram features - Wavelet features - DF	SVM with linear kernel	80	-
[22]	94 CT scans (38 healthy, 23 HCC, 33 metastatic tumors)	- First-order features - GLCM - GLRLM - GLSZM - GLDM - Higher features - Shape features	Naïve Bayes	-	92.68
[23]	435 CT scans	- End-to-end deep learning	Hierarchical multi-modality, multi-scale CNN pipeline	82.5	-
[24]	361 liver DCE-MRI scans	- First-order features - Shape features - LoG features - Wavelet features	SVM	-	85.7
		DenseNet model		-	83.8
[25]	381 liver DCE-MRI scans	- First-order features - GLCM - GLRLM - GLSZM - GLDM - NGTDM - Wavelet features - LoG features - DL features	SVM	93.5	98.8
[37]	392 liver DCE-MRI scans	Visual LI-RADS based features: - Tumor size - Arterial phase hyperenhancement - Washout - Capsule - Mass configuration - Segment location	SVM	82	80
[38]	381 liver DCE-MRI scans	- Self-supervised learning encoder - Liver segmentation using UNet++ - Deep dynamic features - Multi head self-attention for temporal feature modelling	MLP	-	94
Proposed	645 liver DCE-MRI scans	- Kinetic feature - Fractional Zernike–Caputo feature - DF	SVM	94.05	97.21

**Table 6:** Ablation studies.

Experiments	Kernel Filters Ordered According to Convolutional Layers	Accuracy (%)
Ablation study 1	32, 64, and 128	87.35
Ablation study 2	32, 128, and 64	89.20
Ablation study 3	64, 256, and 64	90.11
Ablation study 4	64, 256, and 128	90.75
Ablation study 5	32, 64, 128, and 64	91.60
Ablation study 6	32, 128, 128, and 64	92.00
Ablation study 7	64, 256, 128, and 64	92.25
Ablation study 8	64, 256, 128, and 128	93.10
Ablation study 9	64, 128, 256, and 128	94.05

## Data Availability

The datasets generated or analyzed during the current study are available from the corresponding author upon reasonable request.
